# The activity of the quorum sensing regulator HapR is modulated by the bacterial extracellular vesicle (BEV)‐associated protein ObfA of *Vibrio cholerae*

**DOI:** 10.1002/jev2.12507

**Published:** 2024-09-10

**Authors:** Stephan P. Ebenberger, Fatih Cakar, Yi‐Chi Chen, Katharina Pressler, Leo Eberl, Stefan Schild

**Affiliations:** ^1^ Institute of Molecular Biosciences University of Graz Graz Austria; ^2^ Department of Plant and Microbial Biology University of Zurich Zurich Switzerland; ^3^ BioTechMed‐Graz Graz Austria; ^4^ Field of Excellence Biohealth University of Graz Graz Austria

**Keywords:** bacterial membrane vesicles, biofilm, CsrA, HapR, VC1154, virulence, VPS

## Abstract

*Vibrio cholerae*, a facultative human pathogen and causative agent of the severe diarrheal disease cholera, transits between the human intestinal tract and aquatic reservoirs. Like other bacterial species, *V. cholerae* continuously releases bacterial extracellular vesicles (BEVs) from its surface, which have been recently characterised for their role during in vivo colonisation. However, between epidemic outbreaks, *V. cholerae* persists in the biofilm mode for extended periods in aquatic reservoirs, which enhances environmental fitness and host transition. In this study, we investigated the effect of *V. cholerae* BEVs on biofilm formation, a critical feature for ex vivo survival. In contrast to BEVs from planktonic cultures, our results show that physiological concentrations of BEVs from dynamic biofilm cultures facilitate *V. cholerae* biofilm formation, which could be linked to a proteinaceous factor. Comparative proteomic analyses of planktonic‐ and biofilm‐derived BEVs identified a previously uncharacterised outer membrane protein as an abundant component of dynamic biofilm‐derived BEVs, which was found to be responsible for the BEV‐dependent enhancement of biofilm production. Consequently, this protein was named **o**uter membrane‐associated **b**iofilm **f**acilitating protein **A** (ObfA). Comprehensive molecular studies unravelled ObfA as a negative modulator of HapR activity. HapR is a key transcriptional regulator of the *V. cholerae* quorum sensing (QS) cascade acting as a potent repressor of biofilm formation and virulence. Consistently, *obfA* mutants not only exhibited reduced biofilm production but also reduced colonisation fitness. Surprisingly, our results demonstrate that ObfA does not affect HapR through the canonical QS system but via the Csr‐cascade altering the expression of the small regulatory RNAs CsrC and CsrD. In summary, this study elucidates a novel intraspecies BEV‐based communication in *V. cholerae* that influences biofilm formation and colonisation fitness via a new regulatory pathway involving HapR, Csr‐cascade and the BEV‐associated protein ObfA.

## Graphical abstract

Bacterial extracellular vesicles (BEVs) released by the facultative human pathogen *Vibrio cholerae* during biofilm formation act as an intra‐species communication tool. The BEV‐associated protein ObfA modulates activity of the central cytoplasmic transcriptional regulator HapR and thereby affects biofilm formation as well as virulence, which represent two important stages of the *V. cholerae* lifecycle. ‘Created with BioRender.com’.

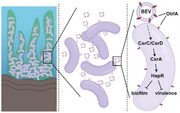

## 1 INTRODUCTION

It is becoming increasingly evident that the production of bacterial extracellular vesicles (BEVs) is not only conserved among bacteria but also plays an important role in the fitness and survival of the donor species. BEVs have been reported to contribute to diverse physiological aspects including nutrient acquisition, disposal of waste products, surface remodelling, transport vehicles and immunomodulation of the host or decoys for membrane‐attacking agents (Toyofuku et al., 2023). Nonetheless, we are at the beginning to understand the versatile functions of BEVs in different bacterial species. In this context, we are studying BEVs derived from the facultative human‐pathogen *Vibrio cholerae*, representing the causative agent of the severe secretory diarrheal disease cholera with an annual global burden of 3–5 million cases and 100,000 deaths (Clemens et al., 2017; Harris et al., 2012).

Infection with *V. cholerae* usually starts with oral ingestion of contaminated food and water. The pathogen passages through the stomach to reach the small intestine representing the primary colonisation site. Upon host entry *V. cholerae* dramatically changes its expression profile to become a potent colonizer of the intestinal tract. This includes the induction of virulence factors, such as the cholera toxin (CT) or the toxin‐coregulated pilus, as well as several changes in its surface, such as altered expression of outer membrane porins and the emergence of lipopolysaccharide modifications, to adapt to the hostile in vivo conditions (Childers & Klose, 2007; Herrera et al., 2014). Due to the activity of the CT, the patient develops severe secretory diarrhoea, which, if left untreated, can lead to acute life‐threatening dehydration, hypovolemic shock and organ failure.

At later stages of the infection, *V. cholerae* changes its gene expression facilitating the pathogen's transition to the next steps of the life cycle (Gumpenberger et al., 2016; Schild et al., 2007). This includes the induction of a so‐called ‘mucosal escape response’ characterised by bacterial detachment from the mucosal surface as well as activation of motility and chemotaxis (Nielsen et al., 2006). Cholera patients can shed enormous amounts of *V. cholerae* as the litres of watery stool produced by a severely affected person typically harbours between 10^7^ and 10^9^ *Vibrios* per litre (Nelson et al., 2009).

Upon release into the aquatic environment, *V. cholerae* again alters its gene expression to adapt to this ex vivo persistence between outbreaks. This stage is characterised by the formation of biofilms, which are multi‐cellular aggregates embedded in an extracellular matrix. Supported by adhesins, such as RbmA, RbmC and Bap1 as well as extracellular DNA, the *Vibrio* polysaccharide (VPS) represents the main structural component of the biofilm matrix (Absalon et al., 2011; Berk et al., 2012; Fong & Yildiz, 2007; Seper et al., 2011; Yildiz & Schoolnik, 1999). VPS accounts for more than 50% of the total mass of the matrix and is required for the structural development of the biofilm (Yildiz & Schoolnik, 1999; Yildiz et al., 2014). Biofilm formation is not only important for the pathogen's environmental persistence but also facilitates host infectivity and thereby promotes transmission of the disease (Colwell et al., 2003; Faruque et al., 2006; Gallego‐Hernandez et al., 2020; Tamayo et al., 2010).

Several adaption processes of *V. cholerae* to diverse conditions along the life cycle are achieved by spatio‐temporal induction of gene expression. For example, virulence factors are controlled by a complex regulatory cascade, also known as the ToxR regulon (Childers & Klose, 2007; DiRita et al., 1991). The genes encoding proteins for VPS synthesis and secretion are arranged in two clusters *vpsA‐K* (VC0917‐27) and *vpsL‐Q* (VC0934‐9), which are positively regulated by the transcriptional regulators VpsR and VpsT (Casper‐Lindley & Yildiz, 2004; Yildiz et al., 2001). Importantly, HapR, the key regulator of the quorum sensing cascade in *V. cholerae*, is an overarching control element modulating the expression of both regulons. HapR acts at high cell density as a negative regulator of biofilm formation and virulence by repression of *vps* genes and substantial parts of the ToxR regulon, respectively (Beyhan et al., 2007; Kovacikova & Skorupski, 2002; Ng & Bassler, 2009; Waters et al., 2008; Yildiz et al., 2004). HapR together with the alternative sigma factor RpoS also coordinates transcriptional changes along the ‘mucosal escape response’ (Nielsen et al., 2006).

Besides defined gene regulation, recent studies suggest that BEVs also contribute to the survival fitness of *V. cholerae* during host colonisation and phage predation (Reyes‐Robles et al., 2018; Zingl et al., 2011, 2020). Like other Gram‐negative bacteria, *V. cholerae* also releases spherical, non‐living nanoparticles from its surface, which are mainly composed of microbial surface components as well as periplasmic content, which is trapped in the lumen of BEVs during the vesiculation process. With BEVs becoming an emerging research recent studies focused on the biogenesis and physiological role of *V. cholerae* BEVs. For example, the small RNA (sRNA) VrrA reduces the expression of the outer membrane protein OmpA, which facilitates BEV release (Song et al., 2008). We recently reported that inactivation or downregulation of the VacJ/Yrb retrograde phospholipid‐transporter system results in elevated release of BEVs in Gram‐negative bacteria, including *V. cholerae* (Roier et al., 2016). Transcriptional silencing of the VacJ/Yrb‐transporter of *V. cholerae* upon entry into the host increases vesiculation in vivo that facilitates bacterial surface exchange and adaptation to the host environment (Zingl et al., 2020). Moreover, BEVs protect the CT from degradation by intestinal proteases and efficiently deliver active toxins to intestinal epithelial cells (Zingl et al., 2021). These studies provide first insights into how BEVs can contribute to the pathophysiology of *V. cholerae* along with infection and intestinal colonisation.

In this study, we aimed to elucidate the impact of BEVs on *V. cholerae* biofilm formation, representing an important stage in the pathogen's life cycle outside of the human host. This study unravels a novel function of *V. cholerae* BEVs in cell‐to‐cell communication. We identified the BEV‐associated protein ObfA and show that it is responsible for BEV‐mediated signalling that silences the activity of the transcriptional regulator HapR. Due to the diverse pathways affected by HapR, ObfA not only modulates biofilm formation but also in vivo colonisation fitness.

## 2 MATERIAL AND METHODS

### 2.1 Bacterial strains and growth conditions

The bacterial strains and plasmids used in this study are listed in Table S1. *V. cholerae* O1 El Tor C6709 was used as the WT strain in all experiments. Unless otherwise noted, strains were grown in Lysogeny Broth (LB, pH 7) at 37°C with aeration (180 rpm) or on LB agar plates at 37°C. Biofilm formation under static conditions at 24°C or under dynamic conditions at room temperature (air‐conditioned room with an ambient temperature ranging from 22°C to 24°C) was achieved with LB (pH 7). Supplements were used at the following final concentrations: streptomycin (Sm), 100 μg/mL; ampicillin (Ap), 100 μg/mL or 50 μg/mL in combination with other antibiotics; chloramphenicol (Cm), 10 μg/mL (*E. coli*) or 2 μg/mL (*V. cholerae*); tetracycline (Tet), 5 μg/mL (*E. coli*) or 0.5 μg/mL (*V. cholerae*); sucrose (10%); glucose (0.2%) and Isopropyl‐β‐thiogalactosid (IPTG 0.5 mM).

### 2.2 Construction of in‐frame deletion mutants, *phoA* reporter strains and expression plasmids

DNA manipulations, such as purifying chromosomal, plasmid or PCR product DNA, performing PCRs, and constructing in‐frame deletion mutants, were carried out as previously described using derivatives of pCVD442 (Leitner et al., 2015; Seper et al., 2011). The study utilised oligonucleotides listed in Table S2. In‐frame deletion mutants were constructed following the method described by Donnenberg and Kaper (1991). Briefly, ∼800 bp PCR fragments located up‐ and down‐stream of the respective gene were amplified using the oligonucleotide pairs X_Y_1 and X_Y_2 as well as X_Y_3 and X_Y_4 (Table S2) where X represents the gene and Y the respective digestion enzyme. After digestion of the PCR fragments with the appropriate restriction enzymes (New England Biolabs) indicated by the name of the oligonucleotide, they were ligated into pCVD442, which was digested with the appropriate restriction enzymes. Unless noted otherwise, ligation products were transformed into DH5αλpir and Ap^R^ colonies were characterised for the correct constructs by PCR (and restriction analysis). Obtained corresponding knockout plasmids are listed in Table S1. To obtain deletion strains generated derivatives of pCVD442 were transformed into *E. coli* Sm10λpir and conjugated into *V. cholerae*. Exconjugants were purified by Sm^R^/Ap^R^ selection. Sucrose selection was used to obtain Ap^S^ colonies and chromosomal deletions/replacements were confirmed by colony PCR.

Derivatives of pGPphoA were constructed to obtain chromosomal transcriptional fusions of *phoA* to respective genes, as the *phoA* acts as a useful genetic marker in *V. cholerae*. Promoterless *phoA* was used for the construction of *obfA*::*phoA, csrA*::*phoA*, *csrB*::*phoA*, *csrC*::*phoA* and *csrD*::*phoA* fusions. The respective gene fragments were amplified by PCR using oligonucleotide pair x::phoA_y_fw and x::phoA_y_rv (Table S2), while x indicates the respective gene, and y represents the restriction site/enzyme. The PCR product was digested with the restriction enzyme indicated by the oligonucleotide name and ligated with similarly digested pGPphoA, resulting in plasmids pGPphoA‐obfA, pGPphoA‐csrA, pGPphoA‐csrB, pGPphoA‐csrC and pGPphoA‐csrD (Table S1). Generated plasmids were first transformed into *E. coli* Sm10λpir and mobilised into the appropriate *V. cholerae* via conjugation. Exconjugants harbouring the chromosomal transcriptional fusion were purified by Sm^R^ and Ap^R^ selection as previously described (Moisi et al., 2009).

For construction of the plasmid pobfA‐FLAG, which expresses *obfA* with C‐terminal FLAG‐tag, the gene was amplified with PCR using the oligonucleotide pairs obfA_EcoRI_fw and obfA_FLAG_XbaI_rv (Table S2). After digestion with EcoRI and XbaI (New England Biolabs) were ligated into a similarly digested pTrc99A vector and transformed into *E. coli* DH5αλpir. The plasmids were isolated and brought into respective *V. cholerae* strains by transformation. Clones were verified with colony PCR.

### 2.3 Isolation of bacterial extracellular vesicles (BEVs)

BEVs were isolated from planktonic, static biofilm and dynamic biofilm conditions resulting in BEVs^PL^, BEVs^sBF^ and BEVs^dBF^ preparations. In general, BEVs^PL^ from planktonic grown culture were isolated as described previously with minor adaptations (Schild et al., 2009). Overnight cultures of the respective *V. cholerae* strains were cultivated in LB at 37°C, adjusted to a starting OD_600_ = 0.01 in fresh LB (pH 7) and grown for 8 h at 37°C and 180 rpm, before the cells were removed from the supernatant by centrifugation (9000 × *g*, 15 min). To obtain BEVs^sBF^ from static biofilm conditions, cultures of the respective *V. cholerae* strains were cultivated in LB at 37°C, adjusted to a starting OD_600_ = 0.01 in fresh LB (pH 7). A total of 100 mL of the inoculated culture were transferred into a 1 L borosilicate bottle and incubated for 30 h at 24°C without shaking resulting in visible biofilm formation on the glass surface (Figure S1a). Multiple bottles were used in parallel to increase the total culture volume. Biofilms were dispersed by rigorous vortexing and pipetting using serological glass pipettes to generate a homogeneous suspension. Previous results indicate that such a mechanical dispersion of *V. cholerae* biofilms does not does not significantly affect cell viability (Seper et al., 2011). Bacterial cells were removed from the supernatant by centrifugation (9000 × *g*, 15 min). To obtain BEVs^dBF^ from dynamically grown biofilms, we used the three‐channel flow cell system as previously described (Pombo et al., 2022; Seper et al., 2011, 2014) with some modifications. Overnight cultures of the respective *V. cholerae* strains were cultivated in LB at 37°C and adjusted to a starting OD_600_ = 0.1 in fresh LB (pH 7). A total of 300 μL of the bacterial suspension was injected into each channel of the flow cell. Multiple three‐channel flow cells were inoculated in parallel to increase culture volume for the isolation of BEVs^dBF^. After 2 h of static incubation, fresh LB (pH 7) was allowed to pass through the flow channels using a 12‐channel peristaltic pump (Watson Marlow 205S) at a constant rate of 3 mL/h resulting in dynamic biofilm formation in the flow cells. The formation of biofilm under these dynamic conditions was confirmed by fluorescence microscopy (Figure S1b, see chapter ‘Visualisation of a dynamic biofilm formation’ for details). Between 14 and 30 h post‐inoculation, the flow‐through containing secreted BEVs and detached cells was collected in sterile glass bottles on ice to minimize degradation and proliferation of detached cells. After 22 and 30 h post‐inoculation, the collected flow‐through was subjected to further purification and later combined to generate the BEVs^dBF^ samples.

To isolate BEVs^PL^, BEVs^sBF^ or BEVs^dBF^ the respective bacterial cell suspension was centrifuged (9000 × *g*, 15 min) to remove bacterial cells. The supernatant was filtered through 0.22 μm pore size filters to remove intact cells. OD_600_ of each culture was determined by photometric measurements using a Beckman Coulter DU730 spectrophotometer for subsequent BEV quantification. To ensure that no bacteria were left in the supernatant, 1 mL of the filtrate was plated on LB‐agar plates and incubated overnight at 37°C. The BEVs present in the filter‐sterilised supernatant were pelleted through subsequent ultracentrifugation (150,000 × *g*, 4°C, 4 h) and resuspended in saline to generate a BEV suspension 1000‐fold concentrated compared to the original culture supernatant. BEVs were stored at −80°C until further use. All BEVs used in this study were characterised by a variety of qualitative and quantitative assays (Table S3), that is, visualisation by TEM (Figure S2), total protein biomass (determined by Bradford), mean and mode particle size (determined by Zetasizer), particle amount (determined by NTA), LPS content (determined by Purpald assay), lipid content (determined by FM 4–64 assay) and nucleic acid content (determined by SYTO 9 staining). Moreover, protein equivalents of the BEV samples were subjected to immunoblot analysis confirming equal presence of the outer membrane protein OmpU (Figure S3), representing a highly abundant protein in BEV samples based on proteomic analyses (Table S4).

### 2.4 Visualisation of a dynamic biofilm formation

For the visualisation of dynamically formed biofilms, the three‐channel flow cell system (DTU Systems Biology, Technical University of Denmark) was inoculated as described above. Briefly, 300 μL of the bacterial suspension [starting OD_600_ = 0.1 in fresh LB (pH 7)] was injected into each channel of the flow cell. After 2 h of static incubation, fresh LB (pH 7) was allowed to pass through the flow channels using a 12‐channel peristaltic pump (Watson Marlow 205S) at a constant rate of 3 mL/h resulting in dynamic biofilm formation in the flow cells. To visualize the biofilm, the dynamic biofilm formed after 30 h was stained with 250 μL of SYTO 9 dye per channel [1:1000 in LB (pH 7)] and incubated for 20 min at RT as previously described (Pombo et al., 2022). Subsequently, images of the biofilm were captured using the inverted microscope Eclipse Ti‐E (Nikon). Excitation was performed at 485 nm, and emission was observed at 498 nm. Images were captured at 5 μm intervals. For the visualisation and processing of image data, the NIS‐Elements BR software (Nikon) was used.

### 2.5 Transmission electron microscopy

To visualize BEVs by transmission electron microscopy (TEM) appropriate dilutions of BEVs in PBS (Sigma–Aldrich) were allowed to adsorb on a pre‐glow‐discharged (15 mA 25 s PELCO easiGlow) formvar‐coated 300‐mesh copper grid (Plano GmbH, SF162‐3). After 1 min, the excess liquid was removed using filter paper. The grid was negatively stained with 1% uranyl acetate for 1 min and dried with filter paper. Samples were visualised using a Zeiss Libra 120 Plus TEM (Carl Zeiss AG, Oberkochen, Germany) and micrographs were recorded with a XF416 4k camera (Tietz Video and Image Processing Systems GmbH, Gauting, Germany).

### 2.6 Protein quantification

Protein concentrations were determined by Bradford assays (Bio‐Rad Laboratories Inc., Protein Assay Dye Reagent) according to the manufacturer's manual as previously described (Thapa et al., 2023). To ensure detection of luminal content of vesicles samples were lysed with 0.1% SDS for 10 min prior to the assay.

### 2.7 Size and nanoparticle amount measurement

Size distributions of the isolated BEVs were assessed by dynamic light scattering (DLS) using the Zetasizer Nano ZS90 (Malvern, UK) as previously described (Zingl et al., 2021). Samples were diluted 1:1000 in saline and processed at 25°C under standard settings (Dispersant Refractive Index = 1.331, viscosity (cP)  =  0.89). Three measurements were performed using a measurement angle of 173° (backscatter), auto measurement duration and ‘seek for optimal position’ as positioning setting. Nanoparticle amounts were evaluated using a NanoSight NS300 instrument (Malvern Panalytical Ltd, UK) with NTA 3.4 software. Samples were diluted in HyClone HyPure Water (Cytiva) to a final concentration of 20–50 particles per frame before being measured in the light scattering mode. For each sample, ten 60 s videos were recorded in the camera level 16 and analysed with processing threshold 5.

### 2.8 3‐Deoxy‐D‐mannooctulosonic acid (KDO) quantification

To quantify the lipopolysaccharide (LPS) content of OMVs, purpald assays were performed as described previously using KDO (Sigma–Aldrich) as a standard (Roier et al., 2016; Zingl et al., 2020). The LPS amounts in the BEVs were quantified by back‐calculation of the respective protein and lipopolysaccharide content, with the results expressed per KDO amount in ng per μg protein.

### 2.9 Lipid quantification

Lipid quantification was performed using FM 4–64 (N‐(3‐Triethylammoniumpropyl)−4‐(6‐(4‐(Diethylamino) Phenyl) Hexatrienyl) Pyridinium Dibromide) as published previously with slight modifications (Vida & Emr, 1995). FM 4–64 was dissolved in PBS to a working solution with a concentration of 0.2 μg/μL. A 4 μL of FM 4–64 solution was mixed in a 96‐well microtiter plate (Greiner Bio‐One, PS, F‐bottom, Black, Cellstar) with 200 μL of BEVs (0.01 μg/μL protein equivalents). BEVs without FM 4–64 dye and FM 4–64 dye‐only samples in PBS were also run as controls. After incubation in the dark at 37°C for 10 min the fluorescence (excitation: 485 nm/emission: 620 nm) was measured in a microplate reader (FLUOstar Omega, BMG LabTech). Data is presented as a ratio calculated as fluorescence (RFU) per μg protein in the BEV sample.

### 2.10 Nucleic acid quantification

Nucleic acid content in BEV samples was quantified by the membrane‐permeable nucleic acid dye SYTO 9. Chromosomal DNA isolated from *V. cholerae* and quantified by Nanodrop 100 (Thermo Scientific) was used as standard in the range of 0–100 ng/μL. A total of 5 μL of an appropriate dilution of the BEV sample was mixed with 40 μL saline and 5 μL of SYTO nine green fluorescent nucleic acid stain (5 mM, Thermo Fisher Scientific) in a 96‐well microtiter plate (Greiner Bio‐One, PS, F‐bottom, Black, Cellstar). After incubation in dark at RT for 1 h the fluorescence (excitation: 485 nm/emission: 520 nm) was measured in a microplate reader (FLUOstar Omega, BMG LabTech). Measured values were used to back‐calculate the nucleic acid concentration of each sample using the standard curve obtained from the chromosomal DNA standard, with the results expressed in nucleic acid amount in ng per μg protein.

### 2.11 Static biofilm assay

Static biofilms in microtiter plates were assayed by crystal violet staining essentially as previously published (Seper et al., 2011; Seper et al., 2014), with some modifications. Briefly, the respective strains were grown overnight in LB, adjusted to an OD_600_ = 0.001 using fresh LB (pH 7) or fresh LB (pH 7) supplemented with BEVs at a final concentration of 0.02 or 1.4 μg/μL protein biomass equivalent. The origin of BEVs is stated together with the respective data sets. Proteinase K‐digested BEVs^dBF^ were acquired as previously described (Thapa et al., 2023). Briefly, BEVs^dBF^ adjusted to 2 μg/μL protein equivalent was incubated overnight at 55°C with 100 μg/mL Proteinase K followed by inactivation for 10 min at 65°C prior to the addition in biofilm assays. Finally, bacterial suspensions adjusted to an OD_600_ = 0.001 were transferred in a 96‐well microtiter plate (U bottom, Sterilin) with 150 μL per well and incubated for 24 or 48 h at 24°C. Wells were subsequently rinsed using a microplate washer (Anthos Mikrosysteme GmbH, Fluido2), biofilm was stained with 0.1% (w/v) crystal violet, solubilised in 96 % (v/v) ethanol and the OD_595_ was measured in a microplate reader (BMG Labtech SPECTROstar^Nano^) to quantify the amount of biofilm.

### 2.12 Preparation of whole cell lysates

Whole cell lysates (WCLs) were obtained from *V. cholerae* cultures grown under specific conditions as indicated. Equal amounts of cells (equivalent to 1.3 mL of OD_600 _= 1) were harvested by centrifugation (5000 × *g*, 5 min) from the respective *V. cholerae* cultures. Cell pellets were directly resuspended in 100 μL SDS‐PAGE sample buffer (Laemmli, 1970), boiled for 30 min and subjected to SDS‐PAGE.

### 2.13 SDS‐PAGE and immunoblot analysis

Proteins were separated by sodium dodecyl sulphate‐polyacrylamide gel electrophoresis (SDS‐PAGE) using polyacrylamide (12%) gels in combination with the Mini‐PROTEAN Tetra cell system (Bio‐Rad, Vienna) (Laemmli, 1970). As molecular mass standard the PageRuler Prestained Protein Ladder 10–180 kDa (Thermo Fisher Scientific) was used as indicated. Subsequently, protein bands were visualised by silver staining or further processed for immunoblot analysis as previously described (Roier et al., 2012; Schild et al., 2008). The anti‐FLAG M2‐HRP monoclonal antibody (A8592, Sigma Aldrich) was used as the sole antibody (1:2000 in 5% BSA/TBS) to detect the FLAG‐tagged ObfA. For detection of OmpU in BEV samples the anti‐OmpU sera (1:500 in 10% skim‐milk/TBS (Leitner et al., 2013)) was used as 1st antibody and HRP‐linked anti‐mouse IgG (1:20,000 in 10% skim‐milk/TBS, Dianova) was used as 2nd antibody. Chemiluminescence detection was performed by using the ECL solution (Clarity Western ECL Blotting Substrates, BIO‐RAD) and subsequent exposure in a ChemiDoc XRS system (Bio‐Rad Laboratories, Inc.) in combination with Quantity One software (Bio‐Rad Laboratories, Inc.).

### 2.14 Silver‐staining

Silver staining was carried out as previously described (Shevchenko et al., 1996; Zingl et al., 2021) for BEVs separated by SDS‐PAGE. After electrophoresis, the gel was fixed in 50% methanol, 5% acetic acid in water overnight. After two washing steps for 20 min with 50% methanol in water and additionally, for 20 s with water, the gel was incubated for 1 min incubation in 0.02% sodium thiosulfate. Afterward, it was washed three times for 20 s in water and incubated in 0.2% silver nitrate solution supplemented with 200 μg/L formaldehyde for 20 min in the dark. The gel was subsequently two times washed for 20 s in water and submerged in 3% sodium carbonate and 200 μg/L formaldehyde until developed to satisfaction. The development was stopped with 1% glycerol for 10 min and then washed with water for 30 min before imaging.

### 2.15 Alkaline phosphatase (PhoA) activity assay

To determine the enzymatic activities for the transcriptional *phoA*‐fusions, alkaline phosphatase assays were performed as described previously (Manoil, 1991). Briefly, *V. cholerae* cultures were cultivated in LB (pH 7) at 24°C. At the given time point bacterial cultures were harvested and subjected to the alkaline phosphatase assays. The activities were expressed in Miller units: (OD_405_ × 1000)/(OD_600_ × 0.96 × ∆t).

### 2.16 Luminescence assay

Respective *V. cholerae* strains carrying the cosmid pBB1 with the *luxCDABE* operon from *V. harveyi* under regulatory control of HapR or the cosmid pBK1003 with a *qrr4‐luxCDABE* promotor fusion (Henke & Bassler, 2004; Svenningsen et al., 2008) were grown overnight in LB, adjusted to an OD_600_ = 0.001 using fresh LB (pH 7) or fresh LB (pH 7) supplemented with BEVs as indicated (final concentration 0.08 μg/μL protein biomass) and inoculated in a black 24‐well plate (24 well Sensoplate, F glass bottom, Greiner Bio‐One) with 1 mL per well. The plate was placed in a microplate reader (FLUOstar Omega, BMG LabTech) at 37°C with aeration achieved by 300 rpm shaking and OD_600_ and bioluminescence were measured every 30 min. The ratio was calculated as follows: Bioluminescence (RLU)/OD_600_.

### 2.17 Fluorescence assay

Respective *V. cholerae* strains carrying the plasmid pLSLS73 with a *hapR‐gfp* fusion (Svenningsen et al., 2008) were grown overnight in LB, adjusted to an OD_600_ = 0.01 using fresh LB (pH 7) and inoculated in a black 24‐well plate (24 well Sensoplate, F glass bottom, Greiner Bio‐One) with 1 mL per well. The plate was placed in a microplate reader (FLUOstar Omega, BMG LabTech) at 37°C with aeration achieved by 300 rpm shaking and OD_600_, and fluorescence (excitation: 485 nm/emission: 520 nm) was measured every 30 min. The ratio was calculated as flowing: fluorescence (RFU)/OD_600_.

### 2.18 Preparation of RNA and qRT‐PCR

To verify the impact of ObfA on *vps* gene expression, the transcription levels were measured by qRT‐PCR in WT and Δ*obfA*. Cultures were inoculated in LB broth at an OD_600_ of 0.02 and grown statically at RT for 24 h. Bacterial RNA extraction, cDNA synthesis and qRT‐PCR were performed as previously published (Seper et al., 2013). Oligonucleotides used for qRT‐PCR are listed in Table S2, labelled as following: qRTPCR_gene_fw and_rv. For each sample, the mean cycle threshold of the test transcript was normalised to the housekeeping gene 16S rRNA and one randomly selected WT reference sample.

### 2.19 Cholera toxin (CT) ELISA

The concentration of CT in cell‐free supernatant samples was quantified using the GM1 ELISA method, as previously described (Vorkapic et al., 2019). Different concentrations of commercially available purified CT (Sigma–Aldrich) in PBS were used as standard. ELISA plates (BRANDplates, 96‐well, immunoGrade) were coated with GM1 ganglioside (10 μg/mL in 60 mM Na_2_CO_3_) overnight. 4% BSA in PBS was used to block the GM1‐coated plates for 1 h at room temperature. Next, 260 μL of supernatant or dilutions thereof in PBS were added to the wells in duplicate and incubated for 1 h at room temperature. Subsequently, a rabbit anti‐CT polyclonal antibody (1:10,000, Sigma‐Aldrich) followed by an HRP‐linked goat anti‐rabbit antibody (1:2000, Dianova) were added to the wells and allowed to incubate for 1 h at room temperature each. For detection of the CT‐antibody complex tetramethylbenzidine (TMB) substrate solution (Thermo Fisher Scientific) was used according to the manufacturers protocol. The colour intensity in each well was measured at 450 nm in a microplate reader (BMG LabTech SPECTROstar^Nano^). CT amount was estimated in the samples by comparison to the standard curve.

### 2.20 Competition assays

Competition assays in infant mice (in vivo assay) or LB were performed with Δ*obfA* competed for ∼22 h against isogenic WT (*lacZ* ^−^) essentially as previously described (Camilli & Mekalanos, 1995; Schild et al., 2007; Zingl et al., 2020). All experiments were conducted in accordance with the rules of the ethics committee at the University of Graz and the corresponding animal protocol, which has been approved by the Austrian Federal Ministry of Science and Research Ref. II/10b (39/12/75ex2017/18). The mice were housed with ad libitum access to food and water and monitored under the care of full‐time staff. Briefly, strains were grown on LB plates overnight, diluted to OD_600_ = 0.002 in a 1:1 ratio, and used to intragastrically inoculate infant mice (5‐ to 6‐day‐old C57BL6). Appropriate dilutions of the inoculum were plated on LB‐Sm/X‐Gal plates to determine the exact input ratio. Approximately 22 h post‐infection mice were euthanised, their small bowels were removed and homogenised in 1 mL of LB with 15% glycerol. In vitro competitions in LB were performed in parallel by inoculation of 2 mL liquid culture with ∼10^5^ CFU from the inoculum and subsequent cultivation for ∼22 h at 37°C with aeration. CFU was determined by plating appropriate dilutions of the homogenised intestine or culture grown in vitro on LB‐Sm/X‐Gal plates. Results are given by the competition index (CI), which is the ratio of *lacZ* ^+^‐CFU to *lacZ* ^−^‐CFU normalised for the input ratio.

### 2.21 Quantitative proteomic analysis

Protein quantification via mass spectrometry (MS) was conducted at the Functional Genomic Center Zurich (FGCZ) at the University of Zurich (UZH). Sample preparation for label‐free quantitative proteomics was done according to the FASP method as described previously (Wisniewski et al., 2009). In brief, 20 μg of protein of each MV sample was quantified using a Qubit Protein Assay kit (Thermo Fisher Scientific). After denaturation of with 4% SDS, 0.1 M DTT, 8 M urea, 100 mM Tris, pH 8.2 at 95°C for 5 min the sample was subjected to IAA alkylation. Trypsin (0.4 μg; Promega) was added overnight and the digested peptides were then acidified by adding trifluoracetic acid to a final concentration of 0.5%. Following desalting with C18 stage tips, the samples were freeze‐dried and stored at −20°C prior to LC‐MS analysis (Rappsilber et al., 2003). For LC‐MS data acquisition, iRT peptides (Biognosys) were added to the samples for calibration. Peptides were separated on an ACQUITY UPLC M‐Class System (Waters) equipped with a HSS T3 C18 reverse‐phase column (1.8 μm, 75 μm × 250 mm, Waters) and analysed by an Orbitrap Fusion Lumos Tribrid mass spectrometer (Thermo Fisher Scientific). Data were acquired with the DDA mode using solvent A (0.1% formic acid in H_2_O) and solvent B (0.1% formic acid in acetonitrile) with a 108 min gradient; 5% B for 3 min, 5%–22% B in 80 min, 22%–32% B in 10 min, 32%–95% B in 5 min, 95% B for 10 min with a column temperature of 50°C and a constant flow rate of 0.3 μL/min. Thermo raw files were converted to the Mascot generic format (MGF) by the Proteome Discoverer (v2.0; Thermo Fisher Scientific) using the automated rule‐based converter control (Barkow‐Oesterreicher et al., 2013). Mascot search was done against the *V. cholerae* O1 proteome database obtained from UniProt combined with common contaminants and decoys with the following parameters: fixed modification carbamidomethyl (C) and variable modification deamidated (NQ) and oxidation (M); max cleavage 1; peptide charges 2^+^, 3^+^, 4^+^; peptide tolerance 10 ppm; MS/MS tolerance 0.6 Da. The GO Annotations Database was used for protein location prediction. Progenesis QI software (v4.2; Waters) was used for quantitative proteomic analysis. Alignment of chromatograms was carried out by combining automatic and manual alignment with iRT peptide standards. Peptides with MS/MS spectra ranking greater than 6 were excluded. Peptide and protein identification was done by Scaffold 5 (Proteome Software) with Mascot searching and was imported back to Progenesis. All experiments were done in three biological replicates. Proteins absent in 2 of 3 replicates for all three BEV types or with peptide counts < 2 were excluded.

### 2.22 Statistical analysis

Unless stated otherwise the data is presented as median with interquartile range (IQR). Statistical differences between data sets were analysed by a Mann‐Whitney U test for a single comparison or a Kruskal–Wallis test followed by Dunn's *post hoc* test in case of multiple comparisons. Differences were considered significant for *p* values of <0.05.

## 3 RESULTS

### 3.1 Biofilm‐derived BEVs facilitate *V. cholerae* biofilm formation via a proteinaceous factor

**FIGURE 1:**
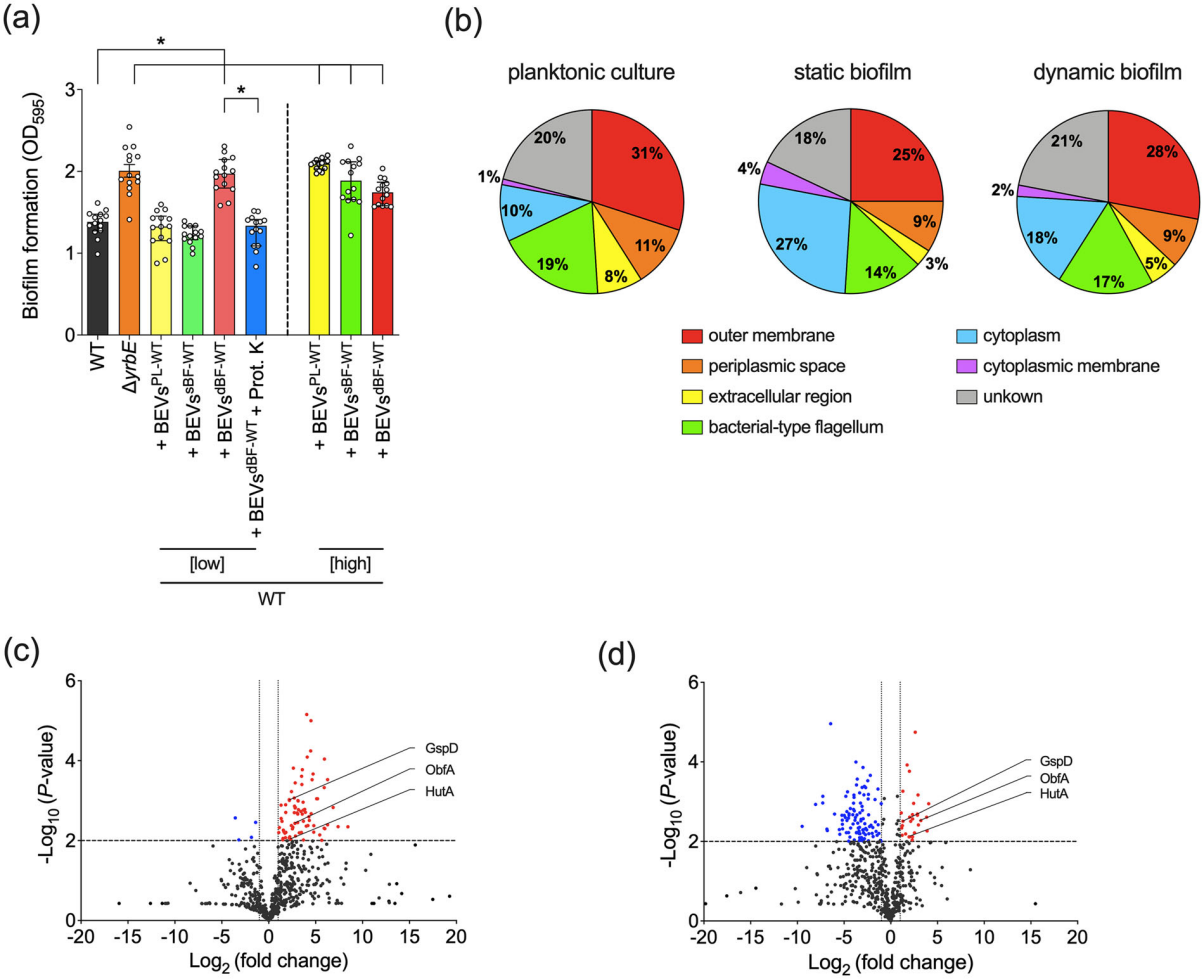
Biofilm‐derived BEVs facilitate biofilm formation in *V. cholerae* via a proteinaceous factor. (a) Biofilms of WT,  Δ*yrbE* and WT supplemented with diverse WT‐derived BEVs as indicated were quantified after 24 h. BEVs were isolated from WT cultures grown under planktonic (+BEVs^PL‐WT^), static biofilm (+BEVs^sBF‐WT^) or dynamic biofilm (+BEVs^dBF‐WT^) conditions and added to the biofilm assays at low (0.02 μg/μL) or high (1.4 μg/μL) concentrations. Additionally, BEVs^dBF‐WT^ digested with proteinase K prior to their addition to biofilm assays were used (BEVs^dBF‐WT^ + Prot. K). The biofilm formation capacity was assayed under static conditions by crystal violet staining and subsequent determination of the OD_595_. Shown are the medians ± interquartile range (IQR) from 14 independent measurements (*n* = 14). An asterisk indicates a significant difference between the data sets (*, *p* < 0.05, using a Kruskal–Wallis test followed by *post hoc* Dunn's multiple comparisons). (b) Pie charts displaying the predicted cellular localization of the top 100 identified proteins of BEVs isolated from WT grown under planktonic (BEVs^PL‐WT^), static biofilm (BEVs^sBF‐WT^) and dynamic biofilm (BEVs^dBF‐WT^) conditions by mass spectrometry (MS) analyses. (c and d) Volcano plots displaying the fold‐change of all 609 proteins identified by MS analyses for the comparison of BEVs^dBF‐WT^ to BEVs^PL‐WT^ (c) and BEVs^dBF‐WT^ to BEVs^sBF‐WT^ (d). Proteins with significantly increased abundance are highlighted by red circles, proteins with significantly decreased abundance are highlighted by blue circles, and proteins without significant changes in abundance are indicated by grey circles. Specifically highlighted are the top three outer membrane proteins with higher abundance in BEVs^dBF‐WT^ compared to BEVs^PL‐WT^ (c) and to BEVs^sBF‐WT^ (d). MS data is provided in the supplementary material (Table S4, *n* = 3 for each BEV type). The statistical analysis employed the two‐stage linear step‐up method of Benjamini, Krieger and Yekutieli with a Q‐value of 1%.

We noticed a significantly increased biofilm formation capacity of the hypervesiculating Δ*yrbE* compared to *V. cholerae* WT (Figure 1a). Intrigued by this observation we isolated *V. cholerae* WT BEVs from planktonic cultures grown to late exponential phase (BEVs^PL‐WT^), from mature static biofilms (BEVs^sBF‐WT^) and from biofilms under dynamic flow conditions (BEVs^dBF‐WT^). It should be noted that during biofilm formation, the dynamic system provides fresh nutrients and removes secreted signalling molecules or metabolic products, resulting in differential gene regulation or importance of genes in the two biofilm models (Muller et al., 2007; Seper et al., 2014). BEVs could be isolated from all three culture conditions, but variations in yield, vesicle diameter, LPS and lipid amount as well as protein profile already indicated defined differences in the BEV composition of BEVs^PL‐WT^, BEVs^sBF‐WT^ and BEVs^dBF‐WT^ (Table S3, Figures S2 and S4). The addition of any of these three BEV types in relatively high amounts, approximately 300–400‐times higher than physiological concentrations, resulted in increased biofilm amounts relative to the WT (Figure 1a). This suggests that BEVs could be a structural component in the biofilm matrix, which is in line with a recent report suggesting that BEVs participate in biofilm matrix assembly (Potapova et al., 2024). However, earlier studies demonstrated that Δ*yrbE* releases only up to 8‐fold more BEVs than the WT (Roier et al., 2016; Zingl et al., 2020). Using such physiologically relevant concentrations, only the addition of BEVs^dBF‐WT^ significantly increased biofilm levels of the WT (Figure 1a). Notably, proteinase K‐treatment of BEVs^dBF‐WT^ prior to their addition in biofilm assays abolished the positive effect on biofilm formation suggesting the involvement of a proteinaceous factor associated with BEVs^dBF‐WT^.

To identify such proteinase K‐accessible proteins, we analysed the proteome BEVs^PL‐WT^, BEVs^sBF‐WT^ and BEVs^dBF‐WT^ by LC‐MS/MS using three biological replicates of each BEV type. This resulted in the identification of 609 proteins of *V. cholerae* in the BEV samples using a cutoff of 2 or more predicted peptides per individual protein (Table S4). We in silico analysed the predicted localisation of the 100 most abundant proteins for BEVs^PL‐WT^, BEVs^sBF‐WT^ and BEVs^dBF‐WT^ using UniProt and PSORTb v3.0 (UniProt, 2023; Yu et al., 2010) (Figure 1b). As expected and consistent with previous BEV proteome data from Gram‐negative bacteria (Altindis et al., 2014; Bhar et al., 2021; Juodeikis et al., 2024; Lappann et al., 2013; Roier et al., 2015), the majority of proteins of all three BEV types could be allocated to the outer membrane (25%–31%), flagellar apparatus (14%–19%), periplasm (9%–11%) and extracellular compartment (3%–8%). Cytoplasmic proteins (10%–27%) and inner membrane (1%–4%) could also be detected, which are generally indications for vesicle formation via explosive cell lysis and not by blebbing from the outer membrane (Toyofuku et al., 2023). Indeed, the highest percentage of the cytoplasmic protein was found in BEVs^sBF‐WT^, which were isolated from stationary batch cultures with cell lysis events accumulating during the 30 h cultivation. Although biofilms readily detached from the glass surface (Figure S1a) and previous studies showed no significant reduction of cell viability upon biofilm dispersion (Seper et al., 2011), cell lysis due to the mechanical disruption of the static biofilms cannot be completely excluded.

Differential normalised abundances between BEVs^dBF‐WT^ and BEVs^PL‐WT^ as well as BEVs^dBF‐WT^ and BEVs^sBF‐WT^ are visualised by volcano plots displaying the ‐log_10_ (*p*‐value) versus log_2_ (fold change) (Figure 1c,d). Points above the non‐axial horizontal line represent proteins with significantly different abundances (*p* < 0.01). Blue points of the left‐most non‐axial vertical line denote proteins, whose normalised abundance is at least 2‐fold decreased in BEVs^dBF‐WT^ compared to BEVs^PL‐WT^ or BEVs^sBF‐WT^. Vice versa, red points of the right‐most non‐axial vertical line highlight proteins, whose normalised abundance is 2‐fold increase in BEVs^dBF‐WT^ compared to BEVs^PL‐WT^ or BEVs^sBF‐WT^. Among the later cohort only three surface proteins, which would be accessible to proteinase K, showed higher normalised abundance in BEVs^dBF‐WT^ compared to BEVs^PL‐WT^ and BEVs^sBF‐WT^. This includes VC1154 (ObfA), VC2733 (GspD) and VCA0576 (HutA). The outer membrane protein GspD is the ‘secretin’ of the type 2 secretion apparatus and the outer membrane protein HutA is involved in heme iron utilisation (Henderson & Payne, 1994; Reichow et al., 2010). VC1154 encodes a 20 kDa hypothetical protein of *V. cholerae* with no annotated function. In silico analyses predicted a beta‐barrel motif frequently found in outer membrane proteins and a low homology to members of the AX21 family with approximately 25% sequence identity. Interestingly, AX21 proteins seem to be associated with BEVs and affect virulence or biofilm formation in other bacteria, such as *Stenotrophomonas maltophilia* and *Xanthomonas oryzae pv. oryzae* (An & Tang, 2018; Bahar et al., 2014, Han et al., 2011). Intrigued by this finding, we focused on VC1154, which we named **o**uter membrane‐associated **b**iofilm **f**acilitating protein **A** (ObfA) based on the functional characterisation of this protein in this study (see below).

### 3.2 BEV‐associated ObfA modulates biofilm formation and *Vibrio* exopolysaccharide expression

**FIGURE 2:**
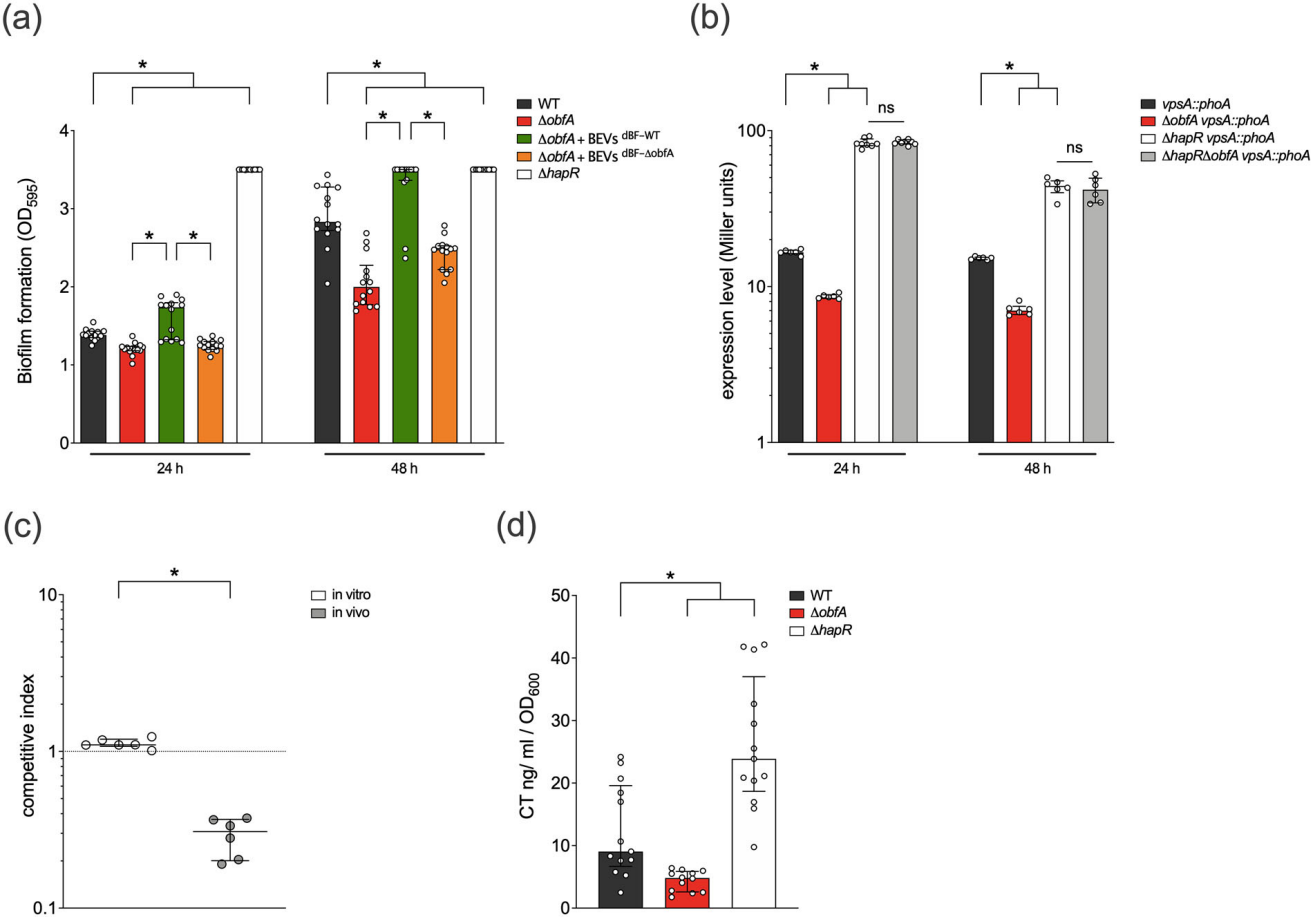
ObfA impacts biofilm formation capacity, vps gene expression, colonisation fitness and cholera toxin (CT) expression. (a) Biofilms of V. cholerae WT, ∆obfA, ∆hapR and ∆obfA supplemented with WT or ∆obfA‐derived BEVs were quantified after 24 and 48 h. BEVs were isolated from WT (+BEVs^dBF‐WT^) or ∆obfA (+BEVs^dBF‐∆obfA^) cultures grown under dynamic biofilm conditions and added the biofilm assays at a final concentration of 0.02 μg/μL. The biofilm formation capacity was assayed under static conditions by crystal violet staining and subsequent determination of the OD_595_. Shown are the medians ± IQR from 14 independent measurements (*n* = 14). An asterisk indicates a significant difference between the data sets (*, *p* < 0.05, using a Kruskal–Wallis test followed by post hoc Dunn's multiple comparisons). (b) Alkaline phosphatase activities (in Miller units) were measured from WT, ∆*obfA*, ∆*hapR* and ∆*obfA*∆*hapR* harbouring a chromosomal *vpsA‐phoA* transcriptional fusion. Cultures were grown at 24°C for 24 and 48 h as indicated. Shown are the medians ± IQR from at least six independent measurements (*n* = 14). An asterisk indicates a significant difference in the data sets, while ns indicates no significant differences (*, *p* < 0.05, using a Kruskal–Wallis test followed by *post hoc* Dunn's multiple comparisons). (c) Results are shown as the competitive index (CI) for competition of ∆*obfA* to a fully virulent LacZ^−^ derivative of the WT (WT*^lacZ−^*) in LB broth (in vitro) and in vivo using the infant mouse model. Each circle represents the CI from a single assay. Horizontal bars indicate the median of each data set (*n* = 6). The asterisks indicate significantly different medians of the in vivo compared to the respective in vitro data set (*, *p* < 0.05, using a Mann‐Whitney U test). (d) Shown is the CT production of *V. cholerae* WT, ∆*obfA* and ∆*hapR* grown under virulence gene factor‐expressing conditions. Shown are the medians ± IQR from at least 12 independent measurements (*n* = 14). An asterisk indicates a significant difference to the WT (*, *p* < 0.05, using a Kruskal–Wallis test followed by *post hoc* Dunn's multiple comparisons).

To analyse the impact of ObfA on biofilm formation, we constructed the non‐polar deletion mutant Δ*obfA* and compared its biofilm formation capacity to the WT. Already at 24 h Δ*obfA* showed a slight, but significant defect in biofilm formation (Figure 2a). This phenotype intensified at 48 h. Expression of *obfA* in trans (∆*obfA* pobfA‐FLAG) significantly restored the biofilm formation capacity compared to a ∆*obfA* mutant carrying the empty vector (∆*obfA* p) (Figure S5a). We also isolated BEVs from ∆*obfA* pobfA‐FLAG as well as the ∆*obfA* p cultures and subjected them to immunoblot analyses. Using an anti‐FLAG antibody, a specific band of approximately 20 kDa for the BEVs derived from ∆*obfA* pobfA‐FLAG, but not for the mutant harbouring the empty vector (Figure S5b). In line with the proteome analyses this result demonstrates that the outer membrane protein ObfA is released from the *V. cholerae* surface via BEVs. For both time points tested, the addition of ObfA‐containing BEVs (BEVs^dBF‐WT^) to ∆*obfA* significantly elevated biofilm formation compared to ∆*obfA* supplemented with BEVs depleted for ObfA (BEV^dBF‐∆^ *^obfA^*) or ∆*obfA* without BEV supplementation (Figure 2a). This demonstrates that externally added ObfA‐containing BEVs are sufficient to restore biofilm formation in ∆*obfA*.

Next, we addressed whether ObfA only acts as a structural component supporting biofilm matrix assembly or also impacts gene expression relevant to the proper biofilm formation of *V. cholerae*. Regarding the latter, differential biofilm formation of *V. cholerae* mutants can be frequently correlated with altered expression of *vps* genes encoding proteins for the *Vibrio* exopolysaccharide (VPS) matrix synthesis and secretion (Yildiz & Schoolnik, 1999). To assess whether *vps* expression is altered in ∆*obfA*, chromosomal transcriptional fusions of a promoterless *phoA* reporter gene to *vpsA* representing one of the first genes in the *vps*‐I locus, were constructed and transferred into the WT and ∆*obfA*, as well as in the ∆*hapR* mutant, which served as a positive control for derepressed *vpsA* expression (Seper et al., 2011). Thus, PhoA activities reflect the transcription levels of *vpsA* in the respective strains. Compared to the WT the ∆*obfA* mutant exhibited significantly lower levels of PhoA activity indicating reduced transcription levels of *vpsA* in ∆*obfA* (Figure 2b). In concordance with the restored biofilm formation of the ∆*obfA* mutant upon addition of ObfA‐containing BEVs^dBF‐WT^ (Figure 2a), the addition of BEVs^dBF‐WT^ to ∆*obfA* resulted in slightly but significantly increased PhoA activities compared to ∆*obfA* supplemented with BEVs depleted for ObfA (BEV^dBF‐∆^ *^obfA^*) (Figure S6). Thus, externally added ObfA‐containing BEVs likely facilitate biofilm formation in ∆*obfA* by stimulating *vps* expression. Expression level analyses by qRT‐PCR confirmed significantly lower transcription of *vpsA* in ∆*obfA* compared to the WT (Figure S7). As reported previously (Seper et al., 2011; Vorkapic et al., 2019), deletion mutants of the quorum sensing (QS) regulator HapR, which is a transcriptional repressor of *vps* genes (Beyhan et al., 2007; Waters et al., 2008; Yildiz et al., 2004), resulted in relatively high *vpsA* expression levels (Figure 2b). Interestingly, the PhoA activity in ∆*hapR*∆*obfA* double mutant was not reduced compared to ∆*hapR*, suggesting that the effect of ObfA on *vps* expression is abrogated in the ∆*hapR* background. This may indicate that ObfA acts via HapR on *vps* expression.

### 3.3 ObfA impacts colonisation fitness and virulence factor expression

Notably, HapR is the key transcription regulator of the QS system in *V. cholerae*, which not only dampens biofilm formation via *vps* gene repression but also impacts colonisation fitness via transcriptional silencing of virulence factor expression (Kovacikova & Skorupski, 2002; Ng & Bassler, 2009). If ObfA influences HapR, a ∆*obfA* mutant should also show altered virulence. To assess colonisation fitness, we conducted competition experiments using the ∆*obfA* against a fully virulent *lacZ* ^−^ derivative of the WT (WT*^lacZ−^*) in LB broth (in vitro) and in vivo using the infant mouse model. Compared with the in vitro control assay, ∆*obfA* showed a significant defect over the WT during intestinal colonisation (Figure 2c). Moreover, we quantified cholera toxin (CT) amounts in the supernatant derived from *V. cholerae* WT and ∆*obfA*, as well as in the ∆*hapR* mutant, which again served as a control for derepressed CT production (Figure 2d). Consistent with the current regulatory model, ∆*hapR* showed significantly higher CT amounts compared to WT. In contrast, ∆*obfA* exhibited significantly lower CT levels than WT. Thus, deletion of *obfA* also affects the pathophysiology of *V. cholerae* by reducing virulence factor expression and colonisation fitness, which reinforces the hypothesis that ObfA acts via HapR.

### 3.4 ObfA modulates HapR activity

We directed our investigation towards the QS system dictating differential *hapR* expression in *V. cholerae*. Like QS regulation in other bacteria, *V. cholerae* senses increasing cell density by extracellular accumulation of continuously released small molecules, also known as autoinducers In *V. cholerae*, autoinducers are recognised by at least four histidine kinase receptors integrating the signal into a cytoplasmic LuxU/O phosphorylation cascade (Miller et al., 2002; Watve et al., 2020). At low cell density, the system ensures phosphorylation of LuxO, which activates the transcription of the four small RNAs (sRNAs) Qrr 1–4 that destabilize the *hapR* mRNA transcript (Lenz et al., 2004). At high cell density, the system drives the dephosphorylation of LuxO, which results in no further activation of the four sRNAs Qrr 1–4. Thus, with increasing cell density the *hapR* mRNA transcript remains stable and ensures rising levels of HapR. As a consequence, HapR levels increase with inclining cell density.

**FIGURE 3:**
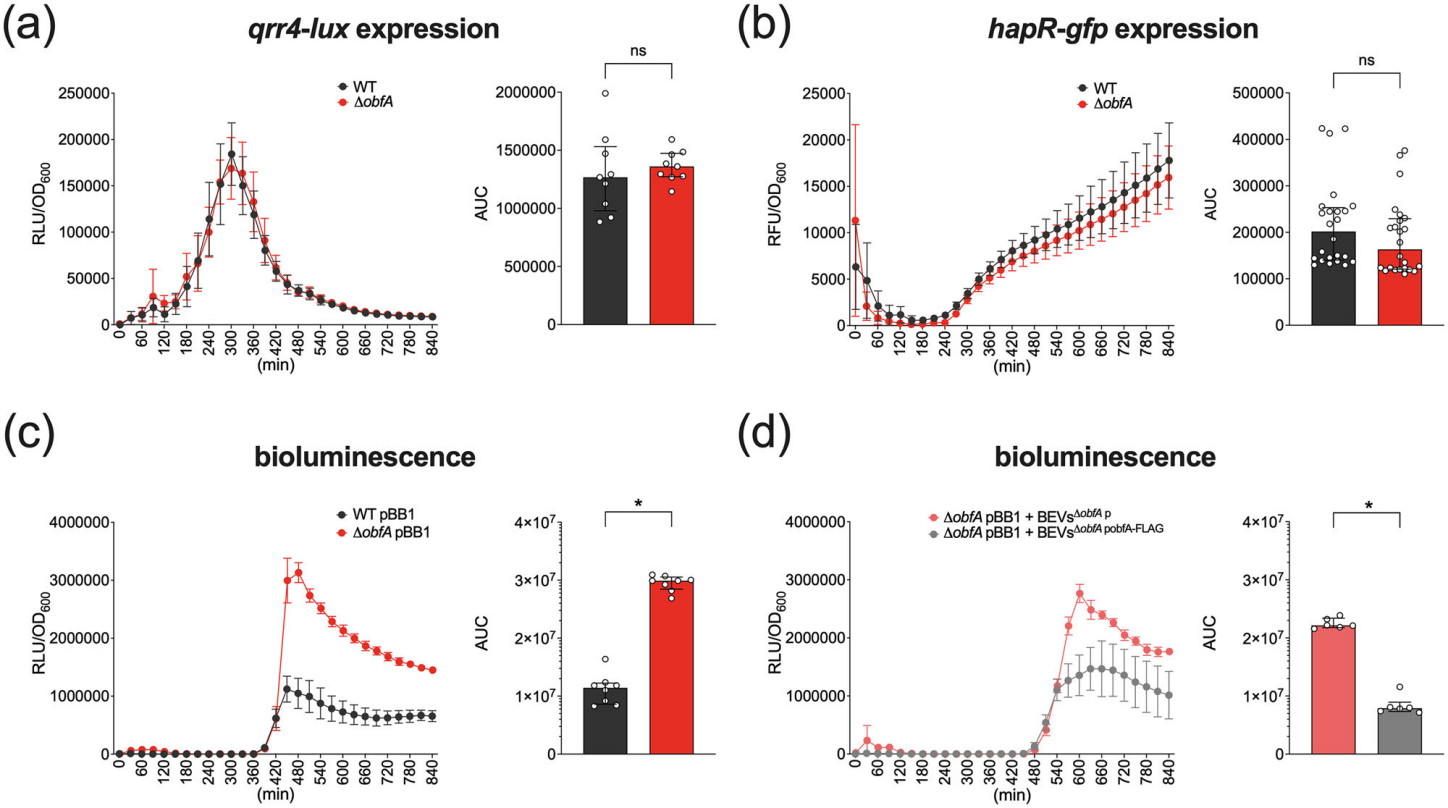
ObfA reduces HapR activity. (a) Light production from the *qrr4*‐*lux* transcription fusion construct was measured in *V. cholerae* WT (black) and ∆*obfA* (red) during growth following inoculation from an overnight culture to a starting OD_600_ of 0.001. The data is presented as median relative light units (RLU) divided by the OD_600_ measured in parallel every 30 min from nine independent experiments (*n* = 9; error bars represent 95% confidence intervals). The bar chart on the right depicts the area under the curve (AUC) ± IQR retrieved from the RLU assays. Statistical analysis revealed no significant difference between the data sets (ns, *p* > 0.05, using a Mann‐Whitney U test). (b) Fluorescence from the *hapR‐gfp* fusion construct indicating HapR expression levels was measured in *V. cholerae* WT (black) and ∆*obfA* (red) during growth following inoculation from an overnight culture to a starting OD_600_ of 0.01. The data is presented as median relative fluorescent units (RFU) divided by the OD_600_ measured in parallel every 30 min from 24 independent experiments (*n* = 24; error bars represent 95% confidence intervals). The bar chart on the right depicts the area under the curve (AUC) ± IQR retrieved from the RFU assays. Statistical analysis revealed no significant difference between the data sets (ns, *p* > 0.05, using a Mann‐Whitney U test). (c) Light production was measured in *V. cholerae* WT (black) and ∆*obfA* (red) carrying the cosmid pBB1 with the HapR‐controlled *luxCDABE* operon during growth following inoculation from an overnight culture to a starting OD_600_ of 0.001. The data is presented as median relative light units (RLU) divided by the OD_600_ measured in parallel every 30 min from eight independent experiments (*n* = 8; error bars represent 95% confidence intervals). The bar chart on the right depicts the area under the curve (AUC) ± IQR retrieved from the RLU assays. An asterisk indicates a significant difference between the data sets (*, *p* < 0.05, using a Mann‐Whitney U test). (d) Light production was measured in ∆*obfA* pBB1 supplemented with BEVs isolated from ∆*obfA* p (red) or with BEVs isolated from ∆*obfA* pobfA‐FLAG (grey) during growth following inoculation from an overnight culture to a starting OD_600_ of 0.001. The data is presented as median relative light units (RLU) divided by the OD_600_ measured in parallel every 30 min from six independent experiments (*n* = 6; error bars represent 95% confidence intervals). The bar chart on the right depicts the area under the curve (AUC) ± IQR retrieved from the RLU assays. An asterisk indicates a significant difference between the data sets (*, *p* < 0.05, using a Mann‐Whitney U test).

First, we used a *qrr4‐luxCDABE* transcriptional fusion construct (pqrr4‐lux) that becomes activated by phosphorylated LuxO at low cell densities and is silenced at higher cell densities. Consequently, a steep rise in luminescence for the WT pqrr4‐lux was observed for approximately 5 h of cultivation followed by a decline due to higher cell densities (Figure 3a). Most importantly, WT pqrr4‐lux and ∆*obfA* pqrr4‐lux showed similar dynamics of luminescence activity excluding that ObfA affects Qrr levels.

Next, we analysed *hapR* expression levels using the *hapR*‐*gfp* fusion construct (phapR‐gfp). As expected, at approximately 5 h a constant increase of fluorescence activity over time could observed for WT phapR‐gfp (Figure 3b). High fluorescence at the beginning of the assays followed by a drop likely arises from high *hapR* expression levels in the relatively dense overnight cultures used as inoculum for the assay. Similar fluorescence dynamics of the WT phapR‐gfp and ∆*obfA* phapR‐gfp suggest that ObfA does not modulate *hapR* expression levels.

Finally, we analysed HapR activity using the HapR‐dependent bioluminescence reporter construct pBB1, which harbours the heterologous *luxCDABE* operon from *V. harveyi* (*luxCDABE_V.h._*). As previously described, the regulators controlling luminescence are functionally conserved between *Vibrio* species, which places the *luxCDABE_V.h._* expression in *V. cholerae* under the control of HapR (Miller et al., 2002). Consistent with the increasing levels of *hapR* transcription along cultivation (Figure 3b), a concomitant increase in luminescence activity of WT pBB1 could be observed (Figure 3c). Notably, the luminescence activity of ∆*obfA* pBB1 remained significantly higher throughout the entire duration of the experiment. As *hapR* transcription levels were not altered in ∆*obfA* (Figure 3b), the high luminescence activity observed for ∆*obfA* pBB1 can only be explained by enhanced HapR activity. To address the impact of ObfA provided externally via BEVs, we added ObfA‐containing BEVs as well as control BEVs, to ∆*obfA* pBB1 in the luminescence HapR activity assays (Figure 3d). Addition of ObfA‐containing BEVs (BEVs^∆^ *^obfA^* ^pobfA‐FLAG^) significantly reduced luminescence activity compared to ∆*obfA* pBB1 supplemented with control BEVs depleted for ObfA (BEVs^∆^ *^obfA^* ^p^) (Figure 3d). Concordant to the restoration of biofilm formation in ∆*obfA* by ObfA‐containing BEVs (Figure 2a), externally added ObfA‐containing BEVs also reduce HapR activity. This suggests that ObfA associated with BEVs can be sensed by *V. cholerae*. Moreover, these results identified BEV‐associated ObfA as a repressive factor of HapR activity independent of the QS system.

### 3.5 ObfA modulates HapR activity via the Csr‐cascade

Besides the QS system regulating *hapR* transcription levels, the VarS/A‐CsrA pathway has been recently reported to affect HapR, in particular by modulating its activity (Tsou et al., 2011). So far identified components of this pathway include a two‐component system comprised of the sensor kinase VarS and the response regulator VarA. Phosphorylated VarA activates transcription of the three sRNAs CsrB, CsrC and CsrD, which decrease the activity of carbon storage regulator A (CsrA) (Lenz et al., 2005). CsrA represents an essential post‐transcriptional regulator for various of regulatory functions, such as carbohydrate metabolism, cell shape, virulence and stimulates HapR activity on the protein level (Jang et al., 2011, 2010; Kamp et al., 2014; Lemos Rocha et al., 2022; Lenz et al., 2005; Mey et al., 2015; Tsou et al., 2011). The generation of *csrA* deletion mutants reportedly failed, which highlights its pivotal role in *V. cholerae*’s viability (Mey et al., 2015).

**FIGURE 4:**
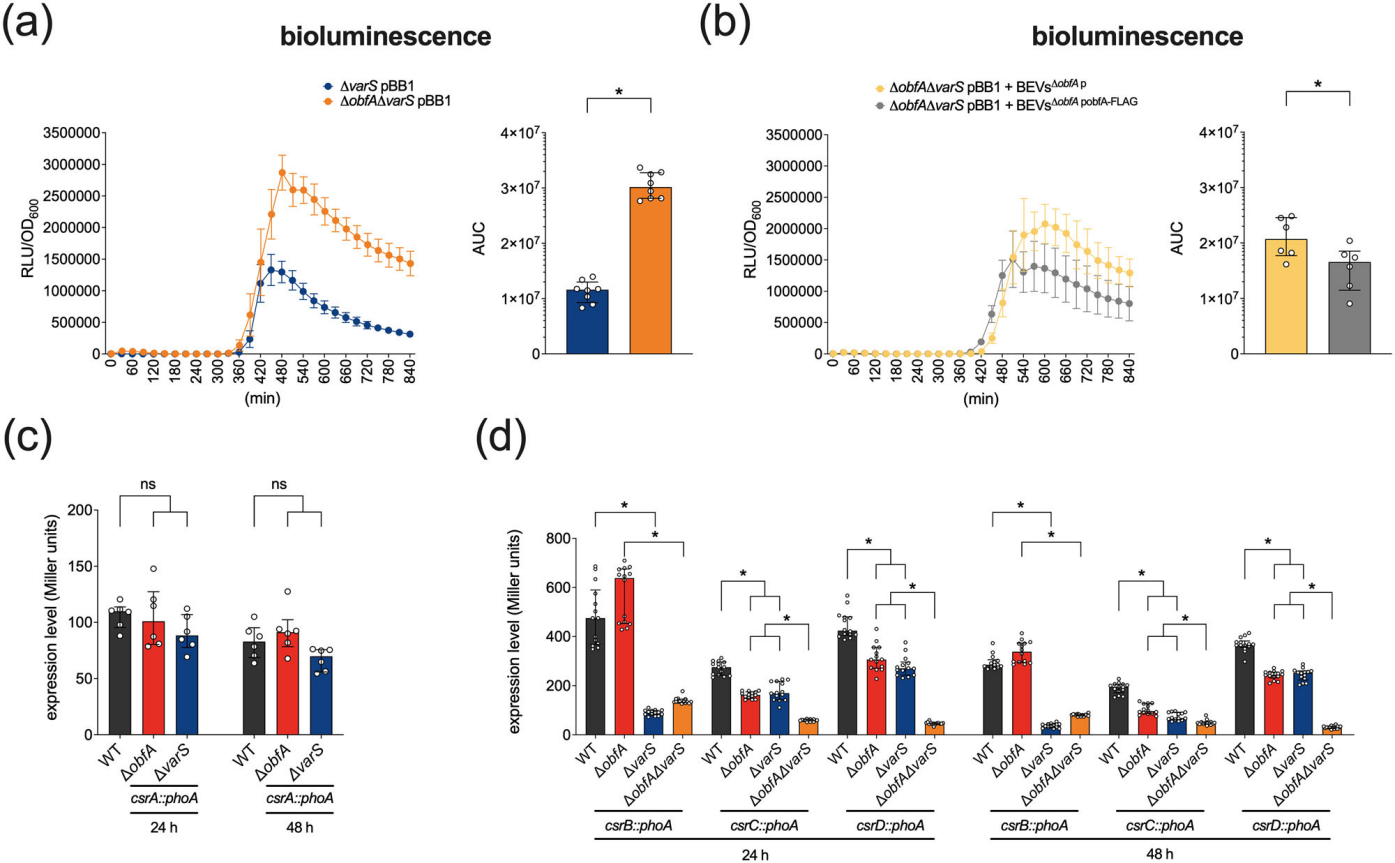
ObfA modulates the CsrA cascade by increasing transcriptional levels of the small RNAs CsrC and CsrD. (a) Light production was measured in ∆*varS* pBB1 (blue) and ∆*obfA*∆*varS* pBB1 (orange) during growth following inoculation from an overnight culture to a starting OD_600_ of 0.001. The data is presented as median relative light units (RLU) divided by the OD_600_ measured in parallel every 30 min from eight independent experiments (*n* = 8; error bars represent 95% confidence intervals). The bar chart on the right depicts the area under the curve (AUC) ± IQR retrieved from the RLU assays. An asterisk indicates a significant difference between the data sets (*, *p* < 0.05, using a Mann‐Whitney U test). (b) Light production was measured in ∆*obfA*∆*varS* pBB1 supplemented with BEVs isolated from ∆*obfA* p (yellow) or BEVs isolated from ∆*obfA* pobfA‐FLAG (grey) during growth following inoculation from an overnight culture to a starting OD_600_ of 0.001. The data is presented as median relative light units (RLU) divided by the OD_600_ measured in parallel every 30 min from six independent experiments (*n* = 6; error bars represent 95% confidence intervals). The bar chart on the right depicts the area under the curve (AUC) ± IQR retrieved from the RLU assays. An asterisk indicates a significant difference between the data sets (*, *p* < 0.05, using a Mann‐Whitney U test). (c) Alkaline phosphatase activities (in Miller Units) were measured from WT (black), ∆*obfA* (red) and ∆*varS* (blue) harbouring a chromosomal *csrA‐phoA* transcriptional fusion. Cultures were grown at 24°C for 24 h (left) and 48 h (right). Shown are the medians ± IQR from six independent measurements (*n* = 6). Statistical analysis revealed no significant difference between the data sets (ns, *p* > 0.05, using a Kruskal–Wallis test followed by *post hoc* Dunn's multiple comparisons). (d) Alkaline phosphatase activities (in Miller Units) were measured from WT (black), ∆*obfA* (red), ∆*varS* (blue) and ∆*obfA*∆*varS* (orange) harbouring a chromosomal *csrB‐phoA*, *csrC‐phoA* or *csrD‐phoA* transcriptional fusion. Cultures were grown at 24°C for 24 h (left) and 48 h (right). Shown are the medians ± IQR from 14 independent measurements (*n* = 14). An asterisk indicates a significant difference of the data sets (*, *p* < 0.05, using a Kruskal–Wallis test followed by *post hoc* Dunn's multiple comparisons).

As the exact signals recognised by VarS are unknown, we first analysed whether ObfA‐dependent modulation of HapR activity is still present in a Δ*varS* background by comparing the luminescence activity of ∆*varS* pBB1 and ∆*obfA*Δ*varS* pBB1 (Figure 4a). As shown above for the WT, deletion of *obfA* in ∆*varS* still results in a significantly higher luminescence levels indicating enhanced HapR activity. Moreover, the addition of ObfA‐containing BEVs (BEVs^∆^ *^obfA^* ^pobfA‐FLAG^) to ∆*obfA*Δ*varS* pBB1 significantly reduced luminescence activity compared to ∆*obfA*Δ*varS* pBB1 supplemented with control BEVs depleted for ObfA (BEVs^∆^ *^obfA^* ^p^) (Figure 4b). Thus, BEV‐associated ObfA modulates HapR activity even in the absence of the sensor kinase VarS. As the current state of knowledge suggests that VarA also receives input from other sensor kinases in addition to VarS, ObfA could affect the VarA‐CsrA pathway without the involvement of VarS (Lenz et al., 2005; Tsou et al., 2011). Thus, we next analysed *csrA* expression levels in WT, Δ*obfA* and Δ*varS* using chromosomal fusion to a promoterless *phoA* reporter gene. Neither Δ*obfA* nor Δ*varS* showed significantly altered PhoA activities compared to the WT indicating stable CsrA expression in all strains tested (Figure 4c). Finally, we investigated the expression of the three sRNAs CsrB, CsrC and CsrD using transcriptional *phoA* reporter gene fusions (Figure 4d). Concordant to earlier reports, transcription levels of all three sRNAs were significantly reduced upon deletion of *varS* (Lenz et al., 2005) for both time points tested. We observed significantly reduced levels of CsrC and CsrD in Δ*obfA* for both time points tested, while CsrB transcription levels in Δ*obfA* remained comparable to the WT. This indicates that in the absence of ObfA transcription of sRNAs CsrC and CsrD is decreased. Based on current literature, this should result in higher CsrA activity and thus increased HapR activity (Butz et al., 2019; Tsou et al., 2011). Consequently, the enhanced HapR activity in Δ*obfA* would more potently silence *vps* and virulence gene expression than in the WT, which is consistent with phenotypes unraveled in this study. Notably, PhoA activities of the Δ*obfA*Δ*varS* double mutant harbouring the *csrC‐phoA* and *csrD*‐*phoA* fusion were significantly reduced compared to the respective single mutants. The additive effect of *obfA* and *varS* mutation on CsrC and CsrD expression underscores that the ObfA signalling pathway operates independently of the sensor kinase VarS.

### 3.6 HapR represses *obfA* expression generating a negative feedback loop

**FIGURE 5:**
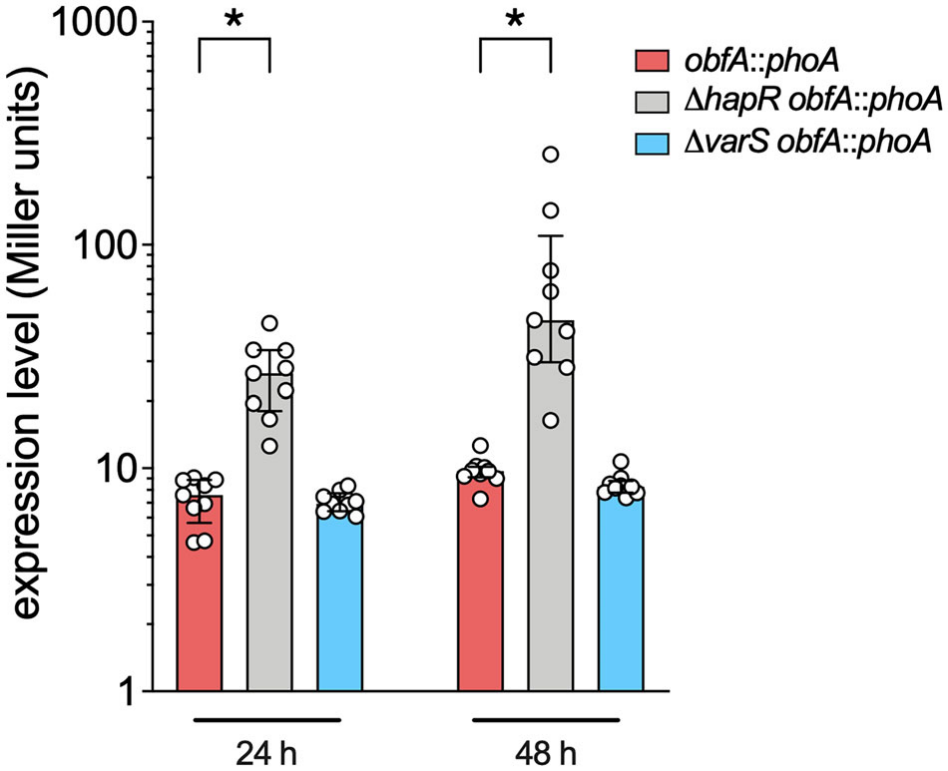
HapR downregulates the expression of obfA. Alkaline phosphatase activities (in Miller units) were measured from WT (red), ∆*hapR* (grey) and ∆*varS* (blue) harbouring a chromosomal *obfA‐phoA* transcriptional fusion. Cultures were grown at 24°C for 24 and 48 h. Shown are the medians ± IQR from nine independent measurements (*n* = 9). An asterisk indicates a significant difference in the data sets (*, *p* < 0.05, using a Mann‐Whitney U test).

The QS system of *V. cholerae* exhibits feedback loops to ensure refined regulation control and accelerate transitions between high and low cell density. For example, HapR can boost the expression of the sRNAs Qrr 1–4, which in turn destabilize *hapR* mRNA resulting in decreased HapR expression (Svenningsen et al., 2008). Moreover, CsrA can activate *varA* expression to increase levels of the sRNA CsrB, CsrC and CsrD, which in turn reduce CsrA activity (Butz et al., 2019). In line with these circuits, HapR or the VarS/A‐CsrA pathway might impact *obfA* expression as ObfA has a negative effect on HapR activity. PhoA activities of a chromosomal *obfA*‐*phoA* transcriptional reporter fusion were not significantly altered in a Δ*varS* mutant compared to the WT (Figure 5). Thus, the VarS/A‐CsrA pathway does not seem to massively affect *obfA* expression. In contrast, PhoA activities of a chromosomal *obfA*‐*phoA* transcriptional reporter fusion were significantly increased in a Δ*hapR* mutant compared to the WT indicating that HapR acts as a repressor for *obfA* expression (Figure 5).

## 4 DISCUSSION

In this study we analysed the impact of *V. cholerae* BEVs on the bacterial pathogen's biofilm formation, representing an important survival strategy during environmental persistence outside of the human host. Besides being a structural component in the biofilm matrix, our results show that physiological concentrations of biofilm‐derived BEVs modulate regulatory pathways in *V. cholerae*. This allowed us to decipher a new intra‐species signalling mechanism via BEVs that depends on the BEV‐associated protein ObfA. Sensing of ObfA‐containing BEVs decreases activity of the transcriptional regulator HapR, a repressor of biofilm formation and virulence (Hammer & Bassler, 2003). Thus, the herein‐identified BEV‐dependent communication affects the aquatic and intestinal life style of *V. cholerae*.

Our results demonstrate that high levels of BEVs can generally facilitate biofilm formation in *V. cholerae*, which is consistent with findings in other Gram‐negative bacteria as well as a recent study on *V. cholerae* biofilm matrix assembly (Baumgarten et al., 2012; Potapova et al., 2024; Yonezawa et al., 2009). This can be most likely attributed to the relatively high abundance of biofilm‐associated proteins, that is, RbmA (VC0928), Bap‐1 (VC1888) and MshA (VC0409), which have been characterised as relevant attachment factors or structural components of the *V. cholerae* biofilm matrix (Absalon et al., 2011; Floyd et al., 2020; Fong et al., 2006; Watnick et al., 1999). Proteomic analyses identified these factors to be among the 100 most abundant proteins in all *V. cholerae* BEV types, i.e. BEVs^PL‐WT^, BEVs^sBF‐WT^ and BEVs^dBF‐WT^. The by far most abundant protein in all three BEV types, however, was the outer membrane protein OmpU (VC0633). A recent study implicated OmpU to govern biofilm matrix assembly in *V. cholerae*, as its absence alters biofilm architecture (Potapova et al., 2024). While ObfA shows the highest abundance in BEVs^dBF‐WT^, it is also present in the other BEV types, that is, BEVs^PL‐WT^ and BEVs^sBF‐WT^. Thus, higher BEV levels in the biofilm assay might result in sufficient incorporation of ObfA‐loaded vesicles irrespective of the BEV type, thereby enhancing biofilm formation via the proposed pathway.

Notably, the effects of bacterial BEVs on biofilms appear to be complex and can differ between species. BEVs do not always promote biofilm formation, but can also inhibit biofilm formation and facilitate biofilm dispersal as shown for BEVs from *Salmonella enterica*, *Yersinia enterocolitica* and *Xylella fastidiosa* (Ionescu et al., 2014; Lu et al., 2020; Ma et al., 2022). BEVs are a substantial component of *Pseudomonas aeruginosa* biofilm matrix (Schooling & Beveridge, 2006). The *Pseudomonas* quinolone signal (PQS), which acts a QS signalling molecule in *P. aeruginosa* and drives the production of BEVs by intercalation into the outer membrane, was reported to promote the formation of mushroom‐shaped mature biofilms (Cooke et al., 2019; Mashburn & Whiteley, 2005; Yang et al., 2009). Moreover, *P. aeruginosa* BEVs contribute to biofilm dispersal in mature biofilms as BEVs in this stage contain substantial amounts of protease, lipases, and nucleases degrading the major matrix components (Cooke et al., 2020). As demonstrated in our study, the dose of BEVs also matters and can mask BEV‐type dependent effects. Thus, the specific impact of bacterial BEVs on biofilms must be comprehensively assessed for each species separately using physiologically relevant doses according to the biofilm model used.

Besides being a structural component in the biofilm matrix, we show that physiological concentrations of biofilm‐derived BEVs modulate regulatory pathways in *V. cholerae*. At this dosage, only BEVs^dBF‐WT^ facilitates biofilm formation by decreasing HapR activity in the bacterial cells, which was attributed to a proteinaceous factor. Indeed, proteome analyses revealed that BEVs released by *V. cholerae* during the planktonic stage, static biofilm formation and dynamic biofilm formation show distinct differences in their composition. Comparative analyses and knock‐out mutagenesis unravelled a regulatory activity of BEVs that is shown to be dependent on the previously uncharacterised BEV‐associated protein ObfA. So far, there are only a few reports that characterised BEVs from other bacteria as a transport system for signalling molecules, which can be recognised by other bacterial cells in the population. For example, PQS is released via BEVs from *P. aeruginosa*, N‐hexadecanoyl‐L‐homoserine lactone is found in *Paracoccus denitrificans* BEVs and the long‐chain ketone CAI‐1 is associated with BEVs from *Vibrio harveyi* (Brameyer et al., 2018; Mashburn & Whiteley, 2005; Toyofuku et al., 2017). Notably, all of these signalling molecules are non‐proteinaceous factors. Thus, the identification of a BEV‐associated protein, that is, ObfA, to play a role in intra‐species communication is an unprecedented finding. To our knowledge, this is also the first report characterizing BEVs as important communication tool for cell‐to‐cell signalling in *V. cholerae*.

**FIGURE 6:**
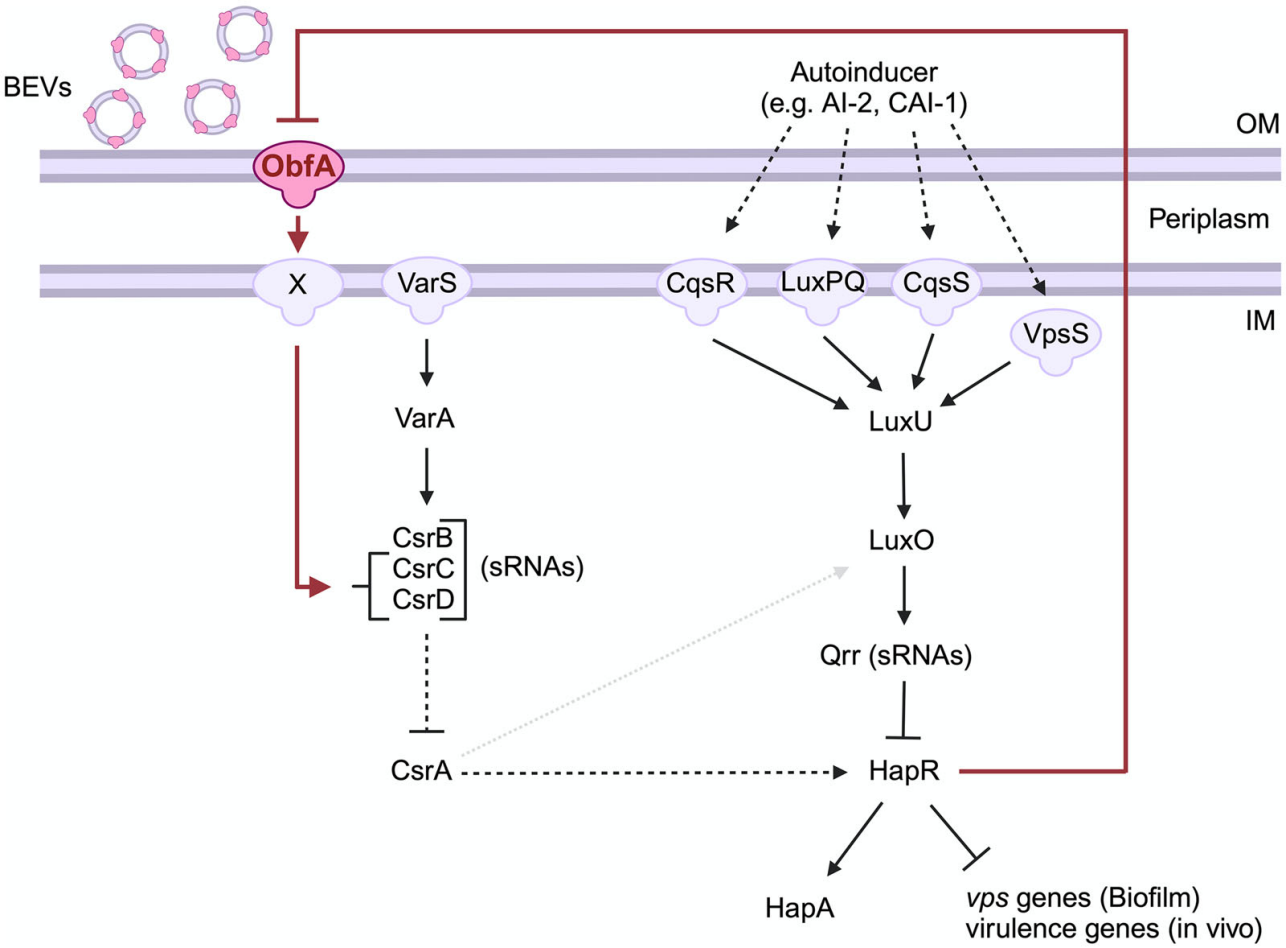
Proposed role of ObfA in the *V. cholerae* quorum sensing network. The previously described pathway is represented in black, while the novel ObfA regulation unravelled in this study is highlighted in red. Transcriptional regulation is indicated as solid lines, while modulation of activity is indicated as dashed lines. ‘Created with BioRender.com’.

Based on the results obtained herein we propose to integrate ObfA as a new player in the HapR regulatory cascade (Figure 6). Absence of ObfA does not alter expression of the sRNA Qrr4 nor HapR. Thus, ObfA is not sensed via the receptors of the canonical QS pathway, that is, CqsR, LuxPQ, CqsS and VpsS, which integrate the incoming signals via LuxU/O to alter Qrr transcription and HapR expression. In line with this result, all identified autoinducer molecules sensed by this pathway are non‐proteinaceous molecules (Higgins et al., 2007; Miller et al., 2002; Watve et al., 2020). In contrast, our data indicate that ObfA modulates the expression of the sRNAs CsrC and CsrD, a function previously attributed to the VarS/A two‐component system (Lenz et al., 2005). As the trigger of the sensor histidine kinase VarS is currently unknown, we thought that ObfA might be sensed via VarS. ObfA‐containing BEVs still decrease HapR activity in a Δ*varS* mutant, although the effect seems less pronounced compared to WT. Moreover, expression levels of CsrC and CsrD in Δ*varS* can be further decreased by additional deletion of *obfA*. The VarS/A system controls the transcription of all three sRNAs CsrB, CsrC and CsrD (Lenz et al., 2005), whereas in a Δ*obfA* mutant, only CsrC and CsrD expression levels are reduced. Thus, VarS does not seem to be the dominant or the only sensor of ObfA. Recent reports suggested that VarA is activated by other, yet to be identified, sensor kinases in addition to VarS (Lenz et al., 2005; Tsou et al., 2011). In *Xanthomonas oryzae pv. oryzae* Ax21‐proteins are recognised by the RaxH/R two‐component system (Han et al., 2011; Lee et al., 2006), but in silico analyses could not identify a closely related homolog in *V. cholerae*. Thus, the exact binding partner of ObfA transferring the signal to the sRNAs remains elusive and its identification needs to be the focus of future endeavours (Figure 6, X). Nonetheless, we have unravelled the downstream effects of ObfA, as expression levels of the sRNAs CsrC and CsrD are altered in an ObfA‐dependent manner. Differential expression levels of the sRNAs affect CsrA activity and consequently HapR activity (Tsou et al., 2011), which explains the phenotypes of the Δ*obfA* mutant as well as the effects upon the addition of ObfA‐containing BEVs observed throughout this study. It should be noted, that CsrA has also been implicated in increased activity of phosphorylated LuxO (Figure 6, grey dotted arrow), which results in enhanced Qrr expression (Lenz et al., 2005). However, this regulation does not appear to play a dominant role within the ObfA circuit or the conditions tested herein as we did not observe any effects of ObfA on Qrr expression. It should also be noted that *V. cholerae* can modulate the decay of the sRNAs CsrB/C/D via MshH, which adds another layer of complexity to this regulatory pathway (Shi et al., 2023).

Why do BEVs^PL‐WT^ and BEVs^sBF‐WT^ show lower ObfA levels than BEVs^dBF‐WT^ and consequently not massively impact to biofilm formation at physiological concentrations? Depending on the growth conditions ObfA might be differentially packed into BEVs or *obfA* expression might be induced under biofilm‐forming conditions by a yet‐to‐be‐identified pathway, which could explain the lower ObfA levels in BEVs^PL‐WT^. Notably, we demonstrate that HapR acts as a repressor on *obfA* transcription generating a negative feedback loop. In the open dynamic flow cell setup fresh nutrients are continuously provided, while waste materials and extracellular signalling molecules are removed. In contrast, the enclosed static biofilm condition as well as the planktonic cultivation allow accumulation of QS autoinducers. Thus, HapR levels in planktonic and static biofilm conditions are rather high resulting in potent repression of *obfA*. This not only explains the lower levels of ObfA in BEVs^sBF‐WT^ compared to BEVs^dBF‐WT^, but also unravels a negative feedback loop as ObfA reduces HapR activity and HapR in turn represses *obfA* transcription. Similar to other regulatory circuits between HapR and the sRNAs Qrr 1–4 or CsrA (Svenningsen et al., 2008). Moreover, CsrA can activate *varA* expression to increase levels of the sRNA CsrB, CsrC and CsrD, which in turn reduce CsrA activity (Butz et al., 2019).

In summary, this study characterizes *V. cholerae* BEVs as an intra‐species communication tool. We identify a novel BEV‐associated protein ObfA, that can modulate the activity of the central cytoplasmic transcriptional regulator HapR and thereby affect biofilm formation as well as colonisation fitness, representing two important pathophysiological aspects of the life cycle of this important facultative human pathogen.

## AUTHOR CONTRIBUTIONS

**Stephan P. Ebenberger**. Formal analysis(Lead); Investigation(Lead); Resources(Equal); Visualization(Lead); Writing—original draft(Supporting); Writing—review & editing(Supporting)**. Fatih Cakar**: Formal analysis (supporting); investigation (supporting); resources (supporting). **Yi‐Chi Chen**: Formal analysis (supporting); investigation (supporting). **Katharina Pressler**: Resources (supporting). **Leo Eberl**: Supervision (supporting); writing—review and editing (supporting). **Stefan Schild**: Conceptualization (lead); funding acquisition (lead); writing—original draft (lead); writing—review and editing (lead).

## CONFLICT OF INTEREST STATEMENT

The authors disclose no conflicts.

## Supporting information



Supporting Information

Supporting Information

## Data Availability

The data that support the findings of this study are available from the corresponding author upon reasonable request.

## References

[jev212507-bib-0001] Absalon, C. , Van Dellen, K. , & Watnick, P. I. (2011). A communal bacterial adhesin anchors biofilm and bystander cells to surfaces. Plos Pathogens, 7, e1002210. 21901100 10.1371/journal.ppat.1002210 PMC3161981

[jev212507-bib-0002] Altindis, E. , Fu, Y. , & Mekalanos, J. J. (2014). Proteomic analysis of Vibrio cholerae outer membrane vesicles. Proceedings of the National Academy of Sciences of the United States of America, 111, E1548–E1556. 24706774 10.1073/pnas.1403683111 PMC3992640

[jev212507-bib-0003] An, S. Q. , & Tang, J. L. (2018). The Ax21 protein influences virulence and biofilm formation in Stenotrophomonas maltophilia. Archives of Microbiology, 200, 183–187. 28965241 10.1007/s00203-017-1433-7 PMC5758655

[jev212507-bib-0004] Bahar, O. , Pruitt, R. , Luu, D. D. , Schwessinger, B. , Daudi, A. , Liu, F. , Ruan, R. , Fontaine‐Bodin, L. , Koebnik, R. , & Ronald, P. (2014). The Xanthomonas Ax21 protein is processed by the general secretory system and is secreted in association with outer membrane vesicles. PeerJ, 2, e242. 24482761 10.7717/peerj.242 PMC3897388

[jev212507-bib-0005] Barkow‐Oesterreicher, S. , Turker, C. , & Panse, C. (2013). FCC—An automated rule‐based processing tool for life science data. Source Code for Biology and Medicine 8, 3. 23311610 10.1186/1751-0473-8-3 PMC3614436

[jev212507-bib-0006] Baumgarten, T. , Sperling, S. , Seifert, J. , von Bergen, M. , Steiniger, F. , Wick, L. Y. , & Heipieper, H. J. (2012). Membrane vesicle formation as a multiple‐stress response mechanism enhances *Pseudomonas putida* DOT‐T1E cell surface hydrophobicity and biofilm formation. Applied and Environmental Microbiology, 78, 6217–6224. 22752175 10.1128/AEM.01525-12 PMC3416621

[jev212507-bib-0007] Berk, V. , Fong, J. C. , Dempsey, G. T. , Develioglu, O. N. , Zhuang, X. , Liphardt, J. , Yildiz, F. H. , & Chu, S. (2012). Molecular architecture and assembly principles of *Vibrio cholerae* biofilms. Science, 337, 236–239. 22798614 10.1126/science.1222981 PMC3513368

[jev212507-bib-0008] Beyhan, S. , Bilecen, K. , Salama, S. R. , Casper‐Lindley, C. , & Yildiz, F. H. (2007). Regulation of rugosity and biofilm formation in Vibrio cholerae: Comparison of VpsT and VpsR regulons and epistasis analysis of vpsT, vpsR, and hapR. Journal of Bacteriology, 189, 388–402. 17071756 10.1128/JB.00981-06 PMC1797413

[jev212507-bib-0009] Bhar, S. , Edelmann, M. J. , & Jones, M. K. (2021). Characterization and proteomic analysis of outer membrane vesicles from a commensal microbe, Enterobacter cloacae. Journal of Proteomics, 231, 103994. 33007464 10.1016/j.jprot.2020.103994

[jev212507-bib-0010] Brameyer, S. , Plener, L. , Muller, A. , Klingl, A. , Wanner, G. , & Jung, K. (2018). Outer membrane vesicles facilitate trafficking of the hydrophobic signaling molecule CAI‐1 between *Vibrio harveyi* cells. Journal of Bacteriology, 200, e00740‐17. 29555694 10.1128/JB.00740-17 PMC6040191

[jev212507-bib-0011] Butz, H. A. , Mey, A. R. , Ciosek, A. L. , & Payne, S. M. (2019). *Vibrio cholerae* CsrA directly regulates *varA* to increase expression of the three nonredundant Csr small RNAs. MBio, 10, 10–1128. 10.1128/mBio.01042-19 PMC6550530 31164471

[jev212507-bib-0012] Camilli, A. , & Mekalanos, J. J. (1995). Use of recombinase gene fusions to identify *Vibrio cholerae* genes induced during infection. Molecular Microbiology, 18, 671–683. 8817490 10.1111/j.1365-2958.1995.mmi_18040671.x PMC4834211

[jev212507-bib-0013] Casper‐Lindley, C. , & Yildiz, F. H. (2004). VpsT is a transcriptional regulator required for expression of *vps* biosynthesis genes and the development of rugose colonial morphology in *Vibrio cholerae* O1 El Tor. Journal of Bacteriology, 186, 1574–1578. 14973043 10.1128/JB.186.5.1574-1578.2004 PMC344397

[jev212507-bib-0014] Childers, B. M. , & Klose, K. E. (2007). Regulation of virulence in *Vibrio cholerae*: The ToxR regulon. Future Microbiology, 2, 335–344. 17661707 10.2217/17460913.2.3.335

[jev212507-bib-0015] Clemens, J. D. , Nair, G. B. , Ahmed, T. , Qadri, F. , & Holmgren, J. (2017). Cholera. Lancet, 390, 1539–1549. 28302312 10.1016/S0140-6736(17)30559-7

[jev212507-bib-0016] Colwell, R. R. , Huq, A. , Islam, M. S. , Aziz, K. M. , Yunus, M. , Khan, N. H. , Mahmud, A. , Sack, R. B. , Nair, G. B. , Chakraborty, J. , Sack, D. A. , & Russek‐Cohen, E. (2003). Reduction of cholera in Bangladeshi villages by simple filtration. Proceedings of the National Academy of Sciences of the United States of America, 100, 1051–1055. 12529505 10.1073/pnas.0237386100 PMC298724

[jev212507-bib-0017] Cooke, A. C. , Florez, C. , Dunshee, E. B. , Lieber, A. D. , Terry, M. L. , Light, C. J. , & Schertzer, J. W. (2020). *Pseudomonas quinolone* signal‐induced outer membrane vesicles enhance biofilm dispersion in *Pseudomonas aeruginosa* . *mSphere*, 5, 10–1128. 10.1128/mSphere.01109-20 PMC7690959 33239369

[jev212507-bib-0018] Cooke, A. C. , Nello, A. V. , Ernst, R. K. , & Schertzer, J. W. (2019). Analysis of *Pseudomonas aeruginosa* biofilm membrane vesicles supports multiple mechanisms of biogenesis. PLoS ONE, 14, e0212275. 30763382 10.1371/journal.pone.0212275 PMC6375607

[jev212507-bib-0019] DiRita, V. J. , Parsot, C. , Jander, G. , & Mekalanos, J. J. (1991). Regulatory cascade controls virulence in *Vibrio cholerae* . Proceedings of the National Academy of Sciences of the United States of America, 88, 5403–5407. 2052618 10.1073/pnas.88.12.5403 PMC51881

[jev212507-bib-0020] Donnenberg, M. S. , & Kaper, J. B. (1991). Construction of an *eae* deletion mutant of enteropathogenic *Escherichia coli* by using a positive‐selection suicide vector. Infection and Immunity, 59, 4310–4317. 1937792 10.1128/iai.59.12.4310-4317.1991 PMC259042

[jev212507-bib-0021] Faruque, S. M. , Biswas, K. , Udden, S. M. , Ahmad, Q. S. , Sack, D. A. , Nair, G. B. , & Mekalanos, J. J. (2006). Transmissibility of cholera: In vivo‐formed biofilms and their relationship to infectivity and persistence in the environment. Proceedings of the National Academy of Sciences of the United States of America, 103, 6350–6355. 16601099 10.1073/pnas.0601277103 PMC1458881

[jev212507-bib-0022] Floyd, K. A. , Lee, C. K. , Xian, W. , Nametalla, M. , Valentine, A. , Crair, B. , Zhu, S. , Hughes, H. Q. , Chlebek, J. L. , Wu, D. C. , Hwan Park, J. , Farhat, A. M. , Lomba, C. J. , Ellison, C. K. , Brun, Y. V. , Campos‐Gomez, J. , Dalia, A. B. , Liu, J. , Biais, N. , … Yildiz, F. H. (2020). c‐di‐GMP modulates type IV MSHA pilus retraction and surface attachment in Vibrio cholerae. Nature Communications, 11, 1549. 10.1038/s41467-020-15331-8 PMC7096442 32214098

[jev212507-bib-0023] Fong, J. C. , Karplus, K. , Schoolnik, G. K. , & Yildiz, F. H. (2006). Identification and characterization of RbmA, a novel protein required for the development of rugose colony morphology and biofilm structure in Vibrio cholerae. Journal of Bacteriology, 188, 1049–1059. 16428409 10.1128/JB.188.3.1049-1059.2006 PMC1347326

[jev212507-bib-0024] Fong, J. C. , & Yildiz, F. H. (2007). The rbmBCDEF gene cluster modulates development of rugose colony morphology and biofilm formation in *Vibrio cholerae* . Journal of Bacteriology, 189, 2319–2330. 17220218 10.1128/JB.01569-06 PMC1899372

[jev212507-bib-0025] Gallego‐Hernandez, A. L. , DePas, W. H. , Park, J. H. , Teschler, J. K. , Hartmann, R. , Jeckel, H. , Drescher, K. , Beyhan, S. , Newman, D. K. , & Yildiz, F. H. (2020). Upregulation of virulence genes promotes *Vibrio cholerae* biofilm hyperinfectivity. Proceedings of the National Academy of Sciences of the United States of America, 117, 11010–11017. 32355001 10.1073/pnas.1916571117 PMC7245069

[jev212507-bib-0026] Gumpenberger, T. , Vorkapic, D. , Zingl, F. G. , Pressler, K. , Lackner, S. , Seper, A. , Reidl, J. , & Schild, S. (2016). Nucleoside uptake in *Vibrio cholerae* and its role in the transition fitness from host to environment. Molecular Microbiology, 99, 470–483. 26202476 10.1111/mmi.13143

[jev212507-bib-0027] Hammer, B. K. , & Bassler, B. L. (2003). Quorum sensing controls biofilm formation in *Vibrio cholerae* . Molecular Microbiology, 50, 101–104. 14507367 10.1046/j.1365-2958.2003.03688.x

[jev212507-bib-0028] Han, S. W. , Lee, S. W. , & Ronald, P. C. (2011). Secretion, modification, and regulation of Ax21. Current Opinion in Microbiology, 14, 62–67. 21236725 10.1016/j.mib.2010.12.006

[jev212507-bib-0029] Harris, J. B. , LaRocque, R. C. , Qadri, F. , Ryan, E. T. , & Calderwood, S. B. (2012). Cholera. Lancet, 379, 2466–2476. 22748592 10.1016/S0140-6736(12)60436-X PMC3761070

[jev212507-bib-0030] Henderson, D. P. , & Payne, S. M. (1994). Characterization of the Vibrio cholerae outer membrane heme transport protein HutA: sequence of the gene, regulation of expression, and homology to the family of TonB‐dependent proteins. Journal of Bacteriology, 176, 3269–3277. 8195082 10.1128/jb.176.11.3269-3277.1994 PMC205497

[jev212507-bib-0031] Henke, J. M. , & Bassler, B. L. (2004). Three parallel quorum‐sensing systems regulate gene expression in Vibrio harveyi. Journal of Bacteriology, 186, 6902–6914. 15466044 10.1128/JB.186.20.6902-6914.2004 PMC522208

[jev212507-bib-0032] Herrera, C. M. , Crofts, A. A. , Henderson, J. C. , Pingali, S. C. , Davies, B. W. , & Trent, M. S. (2014). The *Vibrio cholerae* VprA‐VprB two‐component system controls virulence through endotoxin modification. MBio 5, e02283‐14. 25538196 10.1128/mBio.02283-14 PMC4278540

[jev212507-bib-0033] Higgins, D. A. , Pomianek, M. E. , Kraml, C. M. , Taylor, R. K. , Semmelhack, M. F. , & Bassler, B. L. (2007). The major *Vibrio cholerae* autoinducer and its role in virulence factor production. Nature, 450, 883–886. 18004304 10.1038/nature06284

[jev212507-bib-0034] Ionescu, M. , Zaini, P. A. , Baccari, C. , Tran, S. , da Silva, A. M. , & Lindow, S. E. (2014). Xylella fastidiosa outer membrane vesicles modulate plant colonization by blocking attachment to surfaces. Proceedings of the National Academy of Sciences of the United States of America, 111, E3910–E3918. 25197068 10.1073/pnas.1414944111 PMC4169949

[jev212507-bib-0035] Jang, J. , Jung, K. T. , Park, J. , Yoo, C. K. , & Rhie, G. E. (2011). The *Vibrio cholerae* VarS/VarA two‐component system controls the expression of virulence proteins through ToxT regulation. Microbiology (NY Reading), 157, 1466–1473. 10.1099/mic.0.043737-0 21330435

[jev212507-bib-0036] Jang, J. , Jung, K. T. , Yoo, C. K. , & Rhie, G. E. (2010). Regulation of hemagglutinin/protease expression by the VarS/VarA‐CsrA/B/C/D system in *Vibrio cholerae* . Microbial Pathogenesis, 48, 245–250. 20307644 10.1016/j.micpath.2010.03.003

[jev212507-bib-0037] Juodeikis, R. , Martins, C. , Saalbach, G. , Richardson, J. , Koev, T. , Baker, D. J. , Defernez, M. , Warren, M. , & Carding, S. R. (2024). Differential temporal release and lipoprotein loading in B. thetaiotaomicron bacterial extracellular vesicles. Journal of Extracellular Vesicles, 13, e12406. 38240185 10.1002/jev2.12406 PMC10797578

[jev212507-bib-0038] Kamp, H. D. , Patimalla‐Dipali, B. , Lazinski, D. W. , Wallace‐Gadsden, F. , & Camilli, A. (2014). Gene fitness landscapes of *Vibrio cholerae* at important stages of its life cycle. Plos Pathogens, 9, e1003800. 10.1371/journal.ppat.1003800 PMC3873450 24385900

[jev212507-bib-0039] Kovacikova, G. , & Skorupski, K. (2002). Regulation of virulence gene expression in Vibrio cholerae by quorum sensing: HapR functions at the aphA promoter. Molecular Microbiology, 46, 1135–1147. 12421317 10.1046/j.1365-2958.2002.03229.x

[jev212507-bib-0040] Laemmli, U. K. (1970). Cleavage of structural proteins during the assembly of the head of bacteriophage T4. Nature, 227, 680–685. 5432063 10.1038/227680a0

[jev212507-bib-0041] Lappann, M. , Otto, A. , Becher, D. , & Vogel, U. (2013). Comparative proteome analysis of spontaneous outer membrane vesicles and purified outer membranes of Neisseria meningitidis. Journal of Bacteriology, 195, 4425–4435. 23893116 10.1128/JB.00625-13 PMC3807460

[jev212507-bib-0042] Lee, S. W. , Han, S. W. , Bartley, L. E. , & Ronald, P. C. (2006). From the academy: Colloquium review. Unique characteristics of Xanthomonas oryzae pv. oryzae AvrXa21 and implications for plant innate immunity. Proceedings of the National Academy of Sciences of the United States of America, 103, 18395–18400. 17082309 10.1073/pnas.0605508103 PMC1693675

[jev212507-bib-0043] Leitner, D. R. , Feichter, S. , Schild‐Prufert, K. , Rechberger, G. N. , Reidl, J. , & Schild, S. (2013). Lipopolysaccharide modifications of a cholera vaccine candidate based on outer membrane vesicles reduce endotoxicity and reveal the major protective antigen. Infection and Immunity, 81, 2379–2393. 23630951 10.1128/IAI.01382-12 PMC3697601

[jev212507-bib-0044] Leitner, D. R. , Lichtenegger, S. , Temel, P. , Zingl, F. G. , Ratzberger, D. , Roier, S. , Schild‐Prüfert, K. , Feichter, S. , Reidl, J. , & Schild, S. (2015). A combined vaccine approach against Vibrio cholerae and ETEC based on outer membrane vesicles. Frontiers in Microbiology, 6, 823. 26322032 10.3389/fmicb.2015.00823 PMC4531250

[jev212507-bib-0045] Lemos Rocha, L. F. , Peters, K. , Biboy, J. , Depelteau, J. S. , Briegel, A. , Vollmer, W. , & Blokesch, M. (2022). The VarA‐CsrA regulatory pathway influences cell shape in *Vibrio cholerae* . Plos Genetics, 18, e1010143. 35344548 10.1371/journal.pgen.1010143 PMC8989286

[jev212507-bib-0046] Lenz, D. H. , Miller, M. B. , Zhu, J. , Kulkarni, R. V. , & Bassler, B. L. (2005). CsrA and three redundant small RNAs regulate quorum sensing in *Vibrio cholerae* . Molecular Microbiology, 58, 1186–1202. 16262799 10.1111/j.1365-2958.2005.04902.x

[jev212507-bib-0047] Lenz, D. H. , Mok, K. C. , Lilley, B. N. , Kulkarni, R. V. , Wingreen, N. S. , & Bassler, B. L. (2004). The small RNA chaperone Hfq and multiple small RNAs control quorum sensing in *Vibrio harveyi* and *Vibrio cholerae* . Cell, 118, 69–82. 15242645 10.1016/j.cell.2004.06.009

[jev212507-bib-0048] Lu, J. , Li, L. , Pan, F. , Zuo, G. , Yu, D. , Liu, R. , Fan, H. , & Ma, Z. (2020). PagC is involved in salmonella pullorum OMVs production and affects biofilm production. Veterinary Microbiology, 247, 108778. 32768224 10.1016/j.vetmic.2020.108778

[jev212507-bib-0049] Ma, G. , Ding, Y. , Wu, Q. , Zhang, J. , Liu, M. , Wang, Z. , Wang, Z. , Wu, S. , Yang, X. , Li, Y. , Wei, X. , & Wang, J. (2022). Yersinia enterocolitica‐derived outer membrane vesicles inhibit initial stage of biofilm formation. Microorganisms, 10, 2357. 36557609 10.3390/microorganisms10122357 PMC9786825

[jev212507-bib-0050] Manoil, C. (1991). Analysis of membrane protein topology using alkaline phosphatase and beta‐galactosidase gene fusions. Methods in Cell Biology, 34, 61–75. 1943817 10.1016/s0091-679x(08)61676-3

[jev212507-bib-0051] Mashburn, L. M. , & Whiteley, M. (2005). Membrane vesicles traffic signals and facilitate group activities in a prokaryote. Nature, 437, 422–425. 16163359 10.1038/nature03925

[jev212507-bib-0052] Mey, A. R. , Butz, H. A. , & Payne, S. M. (2015). Vibrio cholerae CsrA regulates ToxR levels in response to amino acids and is essential for virulence. MBio, 6, e01064. 26242626 10.1128/mBio.01064-15 PMC4526715

[jev212507-bib-0053] Miller, M. B. , Skorupski, K. , Lenz, D. H. , Taylor, R. K. , & Bassler, B. L. (2002). Parallel quorum sensing systems converge to regulate virulence in *Vibrio cholerae* . Cell, 110, 303–314. 12176318 10.1016/s0092-8674(02)00829-2

[jev212507-bib-0054] Moisi, M. , Jenul, C. , Butler, S. M. , New, A. , Tutz, S. , Reidl, J. , Klose, K. E. , Camilli, A. , & Schild, S. (2009). A novel regulatory protein involved in motility of Vibrio cholerae. Journal of Bacteriology, 191, 7027–7038. 19767434 10.1128/JB.00948-09 PMC2772493

[jev212507-bib-0055] Muller, J. , Miller, M. C. , Nielsen, A. T. , Schoolnik, G. K. , & Spormann, A. M. (2007). vpsA‐ and luxO‐independent biofilms of Vibrio cholerae. FEMS Microbiology Letters, 275, 199–206. 17697110 10.1111/j.1574-6968.2007.00884.x

[jev212507-bib-0056] Nelson, E. J. , Harris, J. B. , Morris, J. G. Jr. , Calderwood, S. B. , & Camilli, A. (2009). Cholera transmission: The host, pathogen and bacteriophage dynamic. Nature Reviews Microbiology, 7, 693–702. 19756008 10.1038/nrmicro2204 PMC3842031

[jev212507-bib-0057] Ng, W. L. , & Bassler, B. L. (2009). Bacterial quorum‐sensing network architectures. Annual Review of Genetics, 43, 197–222. 10.1146/annurev-genet-102108-134304 PMC4313539 19686078

[jev212507-bib-0058] Nielsen, A. T. , Dolganov, N. A. , Otto, G. , Miller, M. C. , Wu, C. Y. , & Schoolnik, G. K. (2006). RpoS controls the *Vibrio cholerae* mucosal escape response. Plos Pathogens, 2, e109. 17054394 10.1371/journal.ppat.0020109 PMC1617127

[jev212507-bib-0059] Pombo, J. P. , Ebenberger, S. P. , Muller, A. M. , Wolinski, H. , & Schild, S. (2022). Impact of gene repression on biofilm formation of Vibrio cholerae. Frontiers in Microbiology, 13, 912297. 35722322 10.3389/fmicb.2022.912297 PMC9201469

[jev212507-bib-0060] Potapova, A. , Garvey, W. , Dahl, P. , Guo, S. , Chang, Y. , Schwechheimer, C. , Trebino, M. A. , Floyd, K. A. , Phinney, B. S. , Liu, J. , Malvankar, N. S. , & Yildiz, F. H. (2024). Outer membrane vesicles and the outer membrane protein OmpU govern *Vibrio cholerae* biofilm matrix assembly. MBio, 15, e0330423. 38206049 10.1128/mbio.03304-23 PMC10865864

[jev212507-bib-0061] Rappsilber, J. , Ishihama, Y. , & Mann, M. (2003). Stop and go extraction tips for matrix‐assisted laser desorption/ionization, nanoelectrospray, and LC/MS sample pretreatment in proteomics. Analytical Chemistry, 75, 663–670. 12585499 10.1021/ac026117i

[jev212507-bib-0062] Reichow, S. L. , Korotkov, K. V. , Hol, W. G. , & Gonen, T. (2010). Structure of the cholera toxin secretion channel in its closed state. Nature Structural & Molecular Biology, 17, 1226–1232. 10.1038/nsmb.1910 PMC2950906 20852644

[jev212507-bib-0063] Reyes‐Robles, T. , Dillard, R. S. , Cairns, L. S. , Silva‐Valenzuela, C. A. , Housman, M. , Ali, A. , Wright, E. R. , & Camilli, A. (2018). Vibrio cholerae outer membrane vesicles inhibit bacteriophage infection. Journal of Bacteriology, 200, e00792‐17. 29661863 10.1128/JB.00792-17 PMC6040182

[jev212507-bib-0064] Roier, S. , Blume, T. , Klug, L. , Wagner, G. E. , Elhenawy, W. , Zangger, K. , Prassl, R. , Reidl, J. , Daum, G. , Feldman, M. F. , & Schild, S. (2015). A basis for vaccine development: Comparative characterization of Haemophilus influenzae outer membrane vesicles. International Journal of Medical Microbiology, 305, 298–309. 25592265 10.1016/j.ijmm.2014.12.005

[jev212507-bib-0065] Roier, S. , Leitner, D. R. , Iwashkiw, J. , Schild‐Prufert, K. , Feldman, M. F. , Krohne, G. , Reidl, J. , & Schild, S. (2012). Intranasal immunization with nontypeable Haemophilus influenzae outer membrane vesicles induces cross‐protective immunity in mice. PLoS ONE, 7, e42664. 22880074 10.1371/journal.pone.0042664 PMC3411803

[jev212507-bib-0066] Roier, S. , Zingl, F. G. , Cakar, F. , Durakovic, S. , Kohl, P. , Eichmann, T. O. , Klug, L. , Gadermaier, B. , Weinzerl, K. , Prassl, R. , Lass, A. , Daum, G. , Reidl, J. , Feldman, M. F. , & Schild, S. (2016). A novel mechanism for the biogenesis of outer membrane vesicles in Gram‐negative bacteria. Nature Communications, 7, 10515. 10.1038/ncomms10515 PMC4737802 26806181

[jev212507-bib-0067] Schild, S. , Nelson, E. J. , Bishop, A. L. , & Camilli, A. (2009). Characterization of Vibrio cholerae outer membrane vesicles as a candidate vaccine for cholera. Infection and Immunity, 77, 472–484. 19001078 10.1128/IAI.01139-08 PMC2612262

[jev212507-bib-0068] Schild, S. , Nelson, E. J. , & Camilli, A. (2008). Immunization with Vibrio cholerae outer membrane vesicles induces protective immunity in mice. Infection and Immunity, 76, 4554–4563. 18678672 10.1128/IAI.00532-08 PMC2546833

[jev212507-bib-0069] Schild, S. , Tamayo, R. , Nelson, E. J. , Qadri, F. , Calderwood, S. B. , Camilli, A. (2007). Genes induced late in infection increase fitness of *Vibrio cholerae* after release into the environment. Cell Host & Microbe, 2, 264–277. 18005744 10.1016/j.chom.2007.09.004 PMC2169296

[jev212507-bib-0070] Schooling, S. R. , & Beveridge, T. J. (2006). Membrane vesicles: An overlooked component of the matrices of biofilms. Journal of Bacteriology, 188, 5945–5957. 16885463 10.1128/JB.00257-06 PMC1540058

[jev212507-bib-0071] Seper, A. , Fengler, V. H. , Roier, S. , Wolinski, H. , Kohlwein, S. D. , Bishop, A. L. , Camilli, A. , Reidl, J. , & Schild, S. (2011). Extracellular nucleases and extracellular DNA play important roles in *Vibrio cholerae* biofilm formation. Molecular Microbiology, 82, 1015–1037. 22032623 10.1111/j.1365-2958.2011.07867.x PMC3212620

[jev212507-bib-0072] Seper, A. , Hosseinzadeh, A. , Gorkiewicz, G. , Lichtenegger, S. , Roier, S. , Leitner, D. R. , Rohm, M. , Grutsch, A. , Reidl, J. , Urban, C. F. , & Schild, S. (2013). Vibrio cholerae evades neutrophil extracellular traps by the activity of two extracellular nucleases. Plos Pathogens, 9, e1003614. 24039581 10.1371/journal.ppat.1003614 PMC3764145

[jev212507-bib-0073] Seper, A. , Pressler, K. , Kariisa, A. , Haid, A. G. , Roier, S. , Leitner, D. R. , Reidl, J. , Tamayo, R. , & Schild, S. (2014). Identification of genes induced in Vibrio cholerae in a dynamic biofilm system. International Journal of Medical Microbiology, 304, 749–763. 24962154 10.1016/j.ijmm.2014.05.011 PMC4101255

[jev212507-bib-0074] Shevchenko, A. , Wilm, M. , Vorm, O. , & Mann, M. (1996). Mass spectrometric sequencing of proteins silver‐stained polyacrylamide gels. Analytical Chemistry, 68, 850–858. 8779443 10.1021/ac950914h

[jev212507-bib-0075] Shi, M. , Ye, J. , Fan, F. , Zhao, F. , Zhong, X. , Zhong, Z. , Wang, H. , Wang, Z. , & Yang, M. (2023). Precisely controlling Csr sRNA levels by MshH enhances *Vibrio cholerae* colonization in adult mice. Applied and Environmental Microbiology, 89, e0056123. 37404138 10.1128/aem.00561-23 PMC10370335

[jev212507-bib-0076] Song, T. , Mika, F. , Lindmark, B. , Liu, Z. , Schild, S. , Bishop, A. , Zhu, J. , Camilli, A. , Johansson, J. , Vogel, J. , & Wai, S. N. (2008). A new Vibrio cholerae sRNA modulates colonization and affects release of outer membrane vesicles. Molecular Microbiology, 70, 100–111. 18681937 10.1111/j.1365-2958.2008.06392.x PMC2628432

[jev212507-bib-0077] Svenningsen, S. L. , Waters, C. M. , & Bassler, B. L. (2008). A negative feedback loop involving small RNAs accelerates Vibrio cholerae's transition out of quorum‐sensing mode. Genes & Development, 22, 226–238. 18198339 10.1101/gad.1629908 PMC2192756

[jev212507-bib-0078] Tamayo, R. , Patimalla, B. , & Camilli, A. (2010). Growth in a biofilm induces a hyperinfectious phenotype in *Vibrio cholerae* . Infection and Immunity, 78, 3560–3569. 20515927 10.1128/IAI.00048-10 PMC2916270

[jev212507-bib-0079] Thapa, H. B. , Kohl, P. , Zingl, F. G. , Fleischhacker, D. , Wolinski, H. , Kufer, T. A. , & Schild, S. (2023). Characterization of the inflammatory response evoked by bacterial membrane vesicles in intestinal cells reveals an RIPK2‐dependent activation by enterotoxigenic *Escherichia coli* vesicles. Microbiology Spectrum, 11, e0111523. 37306596 10.1128/spectrum.01115-23 PMC10433812

[jev212507-bib-0080] Toyofuku, M. , Morinaga, K. , Hashimoto, Y. , Uhl, J. , Shimamura, H. , Inaba, H. , Schmitt‐Kopplin, P. , Eberl, L. , & Nomura, N. (2017). Membrane vesicle‐mediated bacterial communication. The ISME Journal, 11, 1504–1509. 28282039 10.1038/ismej.2017.13 PMC5437348

[jev212507-bib-0081] Toyofuku, M. , Schild, S. , Kaparakis‐Liaskos, M. , & Eberl, L. (2023). Composition and functions of bacterial membrane vesicles. Nature Reviews Microbiology, 21, 415‐430. 10.1038/s41579-023-00875-5. 36932221

[jev212507-bib-0082] Tsou, A. M. , Liu, Z. , Cai, T. , & Zhu, J. (2011). The VarS/VarA two‐component system modulates the activity of the Vibrio cholerae quorum‐sensing transcriptional regulator HapR. Microbiology (NY Reading), 157, 1620–1628. 10.1099/mic.0.046235-0 PMC3167916 21393367

[jev212507-bib-0083] UniProt, C. (2023). UniProt: The universal protein knowledgebase in 2023. Nucleic Acids Research, 51, D523–D531. 36408920 10.1093/nar/gkac1052 PMC9825514

[jev212507-bib-0084] Vida, T. A. , & Emr, S. D. (1995). A new vital stain for visualizing vacuolar membrane dynamics and endocytosis in yeast. Journal of Cell Biology, 128, 779–792. 7533169 10.1083/jcb.128.5.779 PMC2120394

[jev212507-bib-0085] Vorkapic, D. , Mitterer, F. , Pressler, K. , Leitner, D. R. , Anonsen, J. H. , Liesinger, L. , Mauerhofer, L. M. , Kuehnast, T. , Toeglhofer, M. , Schulze, A. , Zingl, F. G. , Feldman, M. F. , Reidl, J. , Birner‐Gruenberger, R. , Koomey, M. , & Schild, S. (2019). A broad spectrum protein glycosylation system influences type II protein secretion and associated phenotypes in Vibrio cholerae. Frontiers in Microbiology, 10, 2780. 31849912 10.3389/fmicb.2019.02780 PMC6901666

[jev212507-bib-0087] Waters, C. M. , Lu, W. , Rabinowitz, J. D. , & Bassler, B. L. (2008). Quorum sensing controls biofilm formation in *Vibrio cholerae* through modulation of cyclic di‐GMP levels and repression of vpsT. Journal of Bacteriology, 190, 2527–2536. 18223081 10.1128/JB.01756-07 PMC2293178

[jev212507-bib-0088] Watnick, P. I. , Fullner, K. J. , & Kolter, R. (1999). A role for the mannose‐sensitive hemagglutinin in biofilm formation by *Vibrio cholerae* El Tor. Journal of Bacteriology, 181, 3606–3609. 10348878 10.1128/jb.181.11.3606-3609.1999 PMC93833

[jev212507-bib-0089] Watve, S. , Barrasso, K. , Jung, S. A. , Davis, K. J. , Hawver, L. A. , Khataokar, A. , Palaganas, R. G. , Neiditch, M. B. , Perez, L. J. , & Ng, W. L. (2020). Parallel quorum‐sensing system in *Vibrio cholerae* prevents signal interference inside the host. Plos Pathogens, 16, e1008313. 32059031 10.1371/journal.ppat.1008313 PMC7046293

[jev212507-bib-0090] Wisniewski, J. R. , Zougman, A. , Nagaraj, N. , & Mann, M. (2009). Universal sample preparation method for proteome analysis. Nature Methods, 6, 359–362. 19377485 10.1038/nmeth.1322

[jev212507-bib-0091] Yang, L. , Nilsson, M. , Gjermansen, M. , Givskov, M. , & Tolker‐Nielsen, T. (2009). Pyoverdine and PQS mediated subpopulation interactions involved in *Pseudomonas aeruginosa* biofilm formation. Molecular Microbiology, 74, 1380–1392. 19889094 10.1111/j.1365-2958.2009.06934.x

[jev212507-bib-0092] Yildiz, F. , Fong, J. , Sadovskaya, I. , Grard, T. , & Vinogradov, E. (2014). Structural characterization of the extracellular polysaccharide from *Vibrio cholerae* O1 El‐Tor. PLoS ONE, 9, e86751. 24520310 10.1371/journal.pone.0086751 PMC3901696

[jev212507-bib-0093] Yildiz, F. H. , Dolganov, N. A. , & Schoolnik, G. K. (2001). VpsR, a member of the response regulators of the two‐component regulatory systems, is required for expression of vps biosynthesis genes and EPS(ETr)‐associated phenotypes in *Vibrio cholerae* O1 El Tor. Journal of Bacteriology, 183, 1716–1726. 11160103 10.1128/JB.183.5.1716-1726.2001 PMC95057

[jev212507-bib-0094] Yildiz, F. H. , Liu, X. S. , Heydorn, A. , & Schoolnik, G. K. (2004). Molecular analysis of rugosity in a *Vibrio cholerae* O1 El Tor phase variant. Molecular Microbiology, 53, 497–515. 15228530 10.1111/j.1365-2958.2004.04154.x

[jev212507-bib-0095] Yildiz, F. H. , & Schoolnik, G. K. (1999). *Vibrio cholerae* O1 El Tor: Identification of a gene cluster required for the rugose colony type, exopolysaccharide production, chlorine resistance, and biofilm formation. Proceedings of the National Academy of Sciences of the United States of America, 96, 4028–4033. 10097157 10.1073/pnas.96.7.4028 PMC22414

[jev212507-bib-0096] Yonezawa, H. , Osaki, T. , Kurata, S. , Fukuda, M. , Kawakami, H. , Ochiai, K. , Hanawa, T. , & Kamiya, S. (2009). Outer membrane vesicles of *Helicobacter pylori* TK1402 are involved in biofilm formation. BMC Microbiology, 9, 197. 19751530 10.1186/1471-2180-9-197 PMC2749055

[jev212507-bib-0097] Yu, N. Y. , Wagner, J. R. , Laird, M. R. , Melli, G. , Rey, S. , Lo, R. , Dao, P. , Sahinalp, S. C. , Ester, M. , Foster, L. J. , & Brinkman, F. S. (2010). PSORTb 3.0: Improved protein subcellular localization prediction with refined localization subcategories and predictive capabilities for all prokaryotes. Bioinformatics, 26, 1608–1615. 20472543 10.1093/bioinformatics/btq249 PMC2887053

[jev212507-bib-0098] Zingl, F. G. , Kohl, P. , Cakar, F. , Leitner, D. R. , Mitterer, F. , Bonnington, K. E. , Rechberger, G. N. , Kuehn, M. J. , Guan, Z. , Reidl, J. , & Schild, S. (2020). Outer membrane vesiculation facilitates surface exchange and in vivo adaptation of Vibrio cholerae. Cell Host & Microbe, 27, 225–237 e8. 31901519 10.1016/j.chom.2019.12.002 PMC7155939

