# Supplementary material for: The activity of the quorum sensing regulator HapR is modulated by the bacterial extracellular vesicle (BEV)‐associated protein ObfA of Vibrio cholerae
Source: J Extracell Vesicles. 2024 Sep 10;13(9):e12507. doi: 10.1002/jev2.12507 (PMC11386269; doi:10.1002/jev2.12507)
Supplement: Supplementary file 1 — Supporting Information [file JEV2-13-e12507-s002.docx]

**Supporting Information**

**Table S1. Strains and plasmids used in this study**

| **Bacterial strain** | **Description** | **Reference** |
| --- | --- | --- |
| *E.coli* |  |  |
| DH5αλpir | *Escherichia coli*, F^-^ *endA1* *glnV44 thi-1 recA1 relA1 gyrA96 deoR nupG* Φ80∆*lacZ*ΔM15 Δ(*lacZYA*-*argF*) U169 *hsdR17*(_rK_ ^-^ _mK_^+^) λpirRK6 | (1) |
| Sm10λpir | *E. coli,* *thi thr leu tonA lacY supE recA*::RPA-2-Te::Mu λpir, Km^R^ | (1) |
| *V. cholerae* |  |  |
| WT | wild type *V. cholerae* strain serogroup: O1; biotype: El Tor; serotype: Inaba; spontaneous Sm^r^ mutant of C6709, clinical isolate, 1991 Peru, tcpA^+^ctx^+^hapR^+^, Sm^R^ | (2) |
| WT*^lacZ-^* | insertion of res-cassette (res-neo-sacB-res cassette) in *lacZ* of WT, Sm^R^, Km^R^ | (3) |
| ∆*yrbE* | Deletion of *yrbE* in C6709, Sm^R^ | This study |
| ∆*hapR* | Deletion of *hapR* in C6709, Sm^R^ | (4) |
| ∆*obfA* | Deletion of *obfA* in C6709, Sm^R^ | This study |
| ∆*varS* | Deletion of *varS* in C6709, Sm^R^ | This study |
| ∆*obfA*∆*varS* | Deletion of *obfA* and *varS* in C6709, Sm^R^ | This study |
| *vpsA*::pGPphoA | Insertion of pGP*phoA* downstream of *vpsA* in WT C6709, Sm^R^, Ap^R^ | (4) |
| ∆*obfA* *vpsA*::pGPphoA | Insertion of pGPphoA downstream of *vpsA* in Δ*obfA,* Sm^R^, Ap^R^ | This study |
| ∆*hapR* *vpsA*::pGPphoA | Insertion of pGPphoA downstream of *vpsA* in Δ*hapR,* Sm^R^, Ap^R^ | (4) |
| ∆*hapR*∆*obfA* *vpsA*::pGPphoA | Insertion of pGPphoA downstream of *vpsA* in Δ*hapR*Δ*obfA,* Sm^R^, Ap^R^ | This study |
| *obfA*::pGPphoA | Insertion of pGP*phoA* downstream of *obfA* in WT C6709, Sm^R^, Ap^R^ | This study |
| ∆*hapR* *obfA*::pGPphoA | Insertion of pGPphoA downstream of *obfA* in Δ*hapR*, Sm^R^, Ap^R^ | This study |
| *csrA*::pGPphoA | Insertion of pGPphoA downstream of *csrA* in WT C6709, Sm^R^, Ap^R^ | This study |
| *csrB*::pGPphoA | Insertion of pGPphoA downstream of *csrB* in WT C6709, Sm^R^, Ap^R^ | This study |
| *csrC*::pGPphoA | Insertion of pGPphoA downstream of *csrC* in WT C6709, Sm^R^, Ap^R^ | This study |
| *csrD*::pGPphoA | Insertion of pGPphoA downstream of *csrD* in WT C6709, Sm^R^, Ap^R^ | This study |
| ∆*obfA* *csrA*::pGPphoA | Insertion of pGPphoA downstream of *csrA* in Δ*obfA,* Sm^R^, Ap^R^ | This study |
| ∆*obfA* *csrB*::pGPphoA | Insertion of pGPphoA downstream of *csrB* in Δ*obfA,* Sm^R^, Ap^R^ | This study |
| ∆*obfA* *csrC*::pGPphoA | Insertion of pGPphoA downstream of *csrC* in Δ*obfA,* Sm^R^, Ap^R^ | This study |
| ∆*obfA* *csrD*::pGPphoA | Insertion of pGPphoA downstream of *csrD* in Δ*obfA,* Sm^R^, Ap^R^ | This study |
| ∆*varS* *csrA*::pGPphoA | Insertion of pGPphoA downstream of *csrA* in Δ*varS,* Sm^R^, Ap^R^ | This study |
| ∆*varS* *csrB*::pGPphoA | Insertion of pGPphoA downstream of *csrB* in Δ*varS,* Sm^R^, Ap^R^ | This study |
| ∆*varS* *csrC*::pGPphoA | Insertion of pGPphoA downstream of *csrC* in Δ*varS,* Sm^R^, Ap^R^ | This study |
| ∆*varS* *csrD*::pGPphoA | Insertion of pGPphoA downstream of *csrD* in Δ*varS,* Sm^R^, Ap^R^ | This study |
| **Plasmids** | | |
| pCVD442 | *ori6K*, *mobRP4*, *sacB*, Ap^R^ | (5) |
| pCVD∆yrbE | pCVD442 with up- and downstream fragments of *yrbE*, Ap^R^ | **(6)** |
| pCVD∆obfA | pCVD442 with up- and downstream fragments of *obfA*, Ap^R^ | This study |
| pCVD∆varS | pCVD442 with up- and downstream fragments of *varS*, Ap^R^ | This study |
| pqrr4-lux | *qrr4-luxCDABE* promotor fusion, also known as pBK1003 with the V. harveyi luxCDABE locus under regulatory control of *qrr4* promotor*,* obtained from B. Bassler, Cm^R^; | (7) |
| phapR-gfp | Cosmid pLAFR2 expressing a HapR-GFP fusion under control of the *hapR* promotor, also known as pSLS373, obtained from B. Bassler, Tet^R^; | (7) |
| pBB1 | Cosmid pLAFR with V. harveyi luxCDABE locus under regulatory control of HapR, obtained from B. Bassler, Tet^R^ | (8) |
| p | pTrc99A, IPTG-inducible vector, Ap^R^ | **(9)** |
| pobfA-FLAG | pTrc99A expressing ObfA with C-terminal FLAG | This study |
| pGPphoA | pGP704 with promotorless phoA of SM10λpir, Ap^r^ | **(10)** |
| pGPphoA-vpsA | pGPphoA with *vpsA* gene fragment, Ap^R^ | (4) |
| pGPphoA-obfA | pGPphoA with *obfA* gene fragment, Ap^R^ | This study |
| pGPphoA-csrA | pGPphoA with *csrA* gene fragment, Ap^R^ | This study |
| pGPphoA-csrB | pGPphoA with *csrB* gene fragment, Ap^R^ | This study |
| pGPphoA-csrC | pGPphoA with *csrC* gene fragment, Ap^R^ | This study |
| pGPphoA-csrD | pGPphoA with *csrD* gene fragment, Ap^R^ | This study |

**Table S2. Strains and plasmids used in this study.**

| varS_SacI_1 | ATAGAGCTCCGACGGTAGCCTTGATC |
| --- | --- |
| varS_BamHI_2 | TTAGGATCCTATTGTCTAAACATTGTG |
| varS_BamHI_3 | AAAGGATCCCTATCTGAACTGATC |
| varS_XbaI_4 | TATTCTAGAGGGAAAGCTACTATCGC |
| obfA_XbaI_1 | ATTTCTAGATTATGCAACATCAAGGTTGGAT |
| obfA_EcoRI_2 | TTAAGAATTCAATTAAGATAAACCTTTATCTTCTG |
| obfA_EcoRI_3 | ATATGAATTCTCCGTTAGGGTTCGTCGCG |
| obfA_SphI_4 | TAAGCATGCAAGCAAGCTCATACGAACGAT |
| obfA_EcoRI_fw | AAAGAATTCATGCGTAAAACAATCGTTG |
| obfA_FLAG_XbaI_rv | AAATCTAGATCATTTGTCATCGTCGTCCTTGTAGTCGAA  GTTAAAGCGAGCTG |
| obfA::phoA_SacI_fw | ATTGAGCTCTGTCAAACCATGAAC |
| obfA::phoA_KpnI_rv | ATTGGTACCCGACGAACCCTAACG |
| csrA::phoA_SacI_fw | ATTGAGCTCCTACCTTTGAAGTGA |
| csrA::phoA_KpnI_rv | ATTGGTACCCGACCTTTCTTCGAT |
| csrB::phoA_SacI_fw | AAAGAGCTCCATGATGACCAATCTCTTGAA |
| csrB::phoA_KpnI_rv | AAAGGTACCGAGATTCATCTTACTGAGCTG |
| csrC::phoA_SacI_fw | AAAGAGCTCATCTTCATGCTTGCCCTCC |
| csrC::phoA_KpnI_rv | AAAGGTACCAAGAGATAATTTACCCGTTTTG |
| csrD::phoA_SacI_fw | AAAGAGCTCGTAGCCATTGAAGCCAACG |
| csrD::phoA_KpnI_rv | AAAGGTACCGAATTGACTTTACGATATGAGC |
| qRTPCR_vpsA_fw | GTGCAGCAACTCTGTCAAGA |
| qRTPCR_vpsA_rv | AACCACATCTGGCTGTTCAC |
| qRTPCR_16SrRNA_fw | AGGGAGGAAGGTGGTTAAGT |
| qRTPCR_16SrRNA_rv | CGCTACACCTGAAATTCTACCC |

^1^ restriction sites are underlined

**Table S3. Qualitative and quantitative analyses of the isolated BEVs used in this study.**

BEV preparations were analyzed for total protein biomass (determined by Bradford), mean and mode particle size (determined by Zetasizer), particle amount (determined by NTA), LPS content (determined by Purpald assay), lipid content (determined by FM^™^ 4-64 assay) and nucleic acid content (determined by SYTO^™^ 9 staining). BEVs were isolated from *V. cholerae* WT planktonic cultures grown to late exponential phase (BEVs^PL-WT^), from *V. cholerae* WT mature static biofilms (BEVs^sBF-WT^), from *V. cholerae* WT or ∆*obfA* biofilms under dynamic flow conditions (BEVs^dBF-WT^ or BEVs^dBF-∆^*^obfA^*) as well as ∆*obfA* p and ∆*obfA* pobfA-FLAG planktonic cultures grown to late exponential phase with 0.5 mM IPTG. Due to the isolation process the BEV preparations are 1000-fold concentrated compared to the original concentration in the bacterial culture supernatant. Data is presented as mean ± standard deviation (n=3).

| **Bacterial membrane vesicles (BEVs)** | **protein amount (µg µl^-1^)** | **mean**  **size**  **(nm)** | **mode**  **size**  **(nm)** | **particle amount**  **(per μg protein equivalent)** | **nucleic acid amount**  **(ng per μg protein equivalent)** | **KDO amount**  **(ng per μg protein equivalent)** | **RFU**  **(per μg protein equivalent)** |
| --- | --- | --- | --- | --- | --- | --- | --- |
| BEVs^PL-WT^ | 6.9 ± 0.9 | 90.4 ± 1.2 | 121.1 ± 10.3 | 1.37 x 10^9^ ± 3.28 x 10^8^ | 71.3 ± 4.6 | 74.2 ± 10.1 | 4.01 x10^6^ ± 5.28 x 10^4^ |
| BEVs^sBF-WT^ | 11.5 ± 1.4 | 70.9 ± 1.9 | 82.1 ± 1.9 | 6.74 x 10^8^ ± 9.45 x 10^7^ | 99.8 ± 5.1 | 73.2 ± 5.8 | 1.33 x 10^6^ ± 5.17 x 10^4^ |
| BEVs^dBF-WT^ | 2.6 ± 0.3 | 60.3 ± 2.6 | 60.8 ± 1.1 | 8.77 x 10^8^ ± 1.41 x 10^8^ | 50.2 ± 1.9 | 87.5 ± 2.5 | 1.64 x 10^5^ ± 7.91 x 10^4^ |
| BEVs^dBF-∆^*^obfA^* | 6.6 ± 0.1 | 58.6 ± 4.0 | 62.1 ± 2.6 | 6.70 x 10^7^ ± 4.33 x 10^6^ | 32.4 ± 1.3 | 144.1 ± 3.8 | 4.32 x 10^5^ ± 2.40 x 10^4^ |
| BEVs^∆^*^obfA^* ^p^ | 4.0 ± 0.7 | 44.5 ± 0.3 | 49.9 ± 0.8 | 1.06 x 10^9^ ± 1.23 x 10^8^ | 95.9 ± 4. | 169.8 ± 9.4 | 2.50 x 10^6^ ± 1.15 x 10^5^ |
| BEVs^∆^*^obfA^* ^pobfA-FLAG^ | 6.9 ± 2.1 | 52.6 ± 0.7 | 58.7 ± 2.1 | 6.69 x 10^8^ ± 1.17 x 10^8^ | 123.1 ± 5.9 | 182.7 ± 2.5 | 1.54 x 10^6^ ± 6.68 x 10^4^ |

*
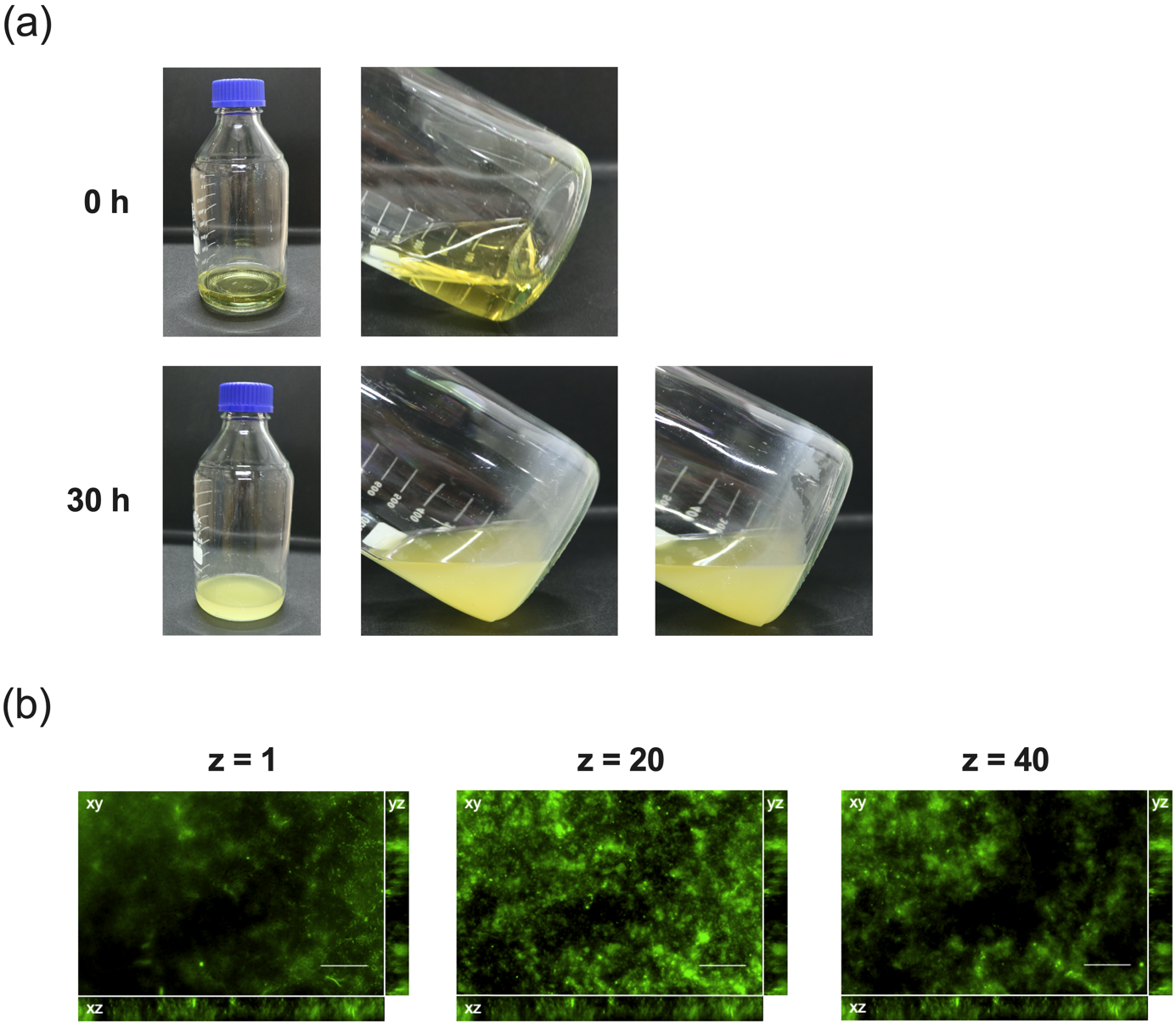
*

**Figure S1: Visualization of static- and dynamic biofilm formation of *V. cholerae* WT cultures used for BEV isolation.**

**(a)** The representative images visualize the static biofilm formation within a 1 L bottle inoculated with *V. cholerae* WT adjusted to a starting OD_600_ = 0.01 in 100 mL LB (pH 7). The upper row shows the 1 L bottle at starting time point (0 h), the lower row incubation for 30 h at 24°C without shaking. From left to right: Images on the far right show the bottles in upright position used for incubation. Images on the right (t =0) or in the middle (t = 30 h) show the bottle in an angled position for illustrative purposes of biofilm formation. Biofilm formed after 30 h is visible as white smear on the inner side of the bottle. The image on the far right in the lower row (t = 30 h) was taken approximately 1 min later already showing visible detachment of the biofilm from the glass surface.

**(b)** Fluorescent microscopy images of SYTO^™^ 9-stained biofilms as horizontal (xy) and vertical (xz and yz) projections (large and side panels, respectively) of WT. The biofilm matured for 30 h in LB (pH 7) under constant continuous flow of fresh medium. Optical sectioning was performed in 0.5 μm steps. Scale bar = 50 μm.

**
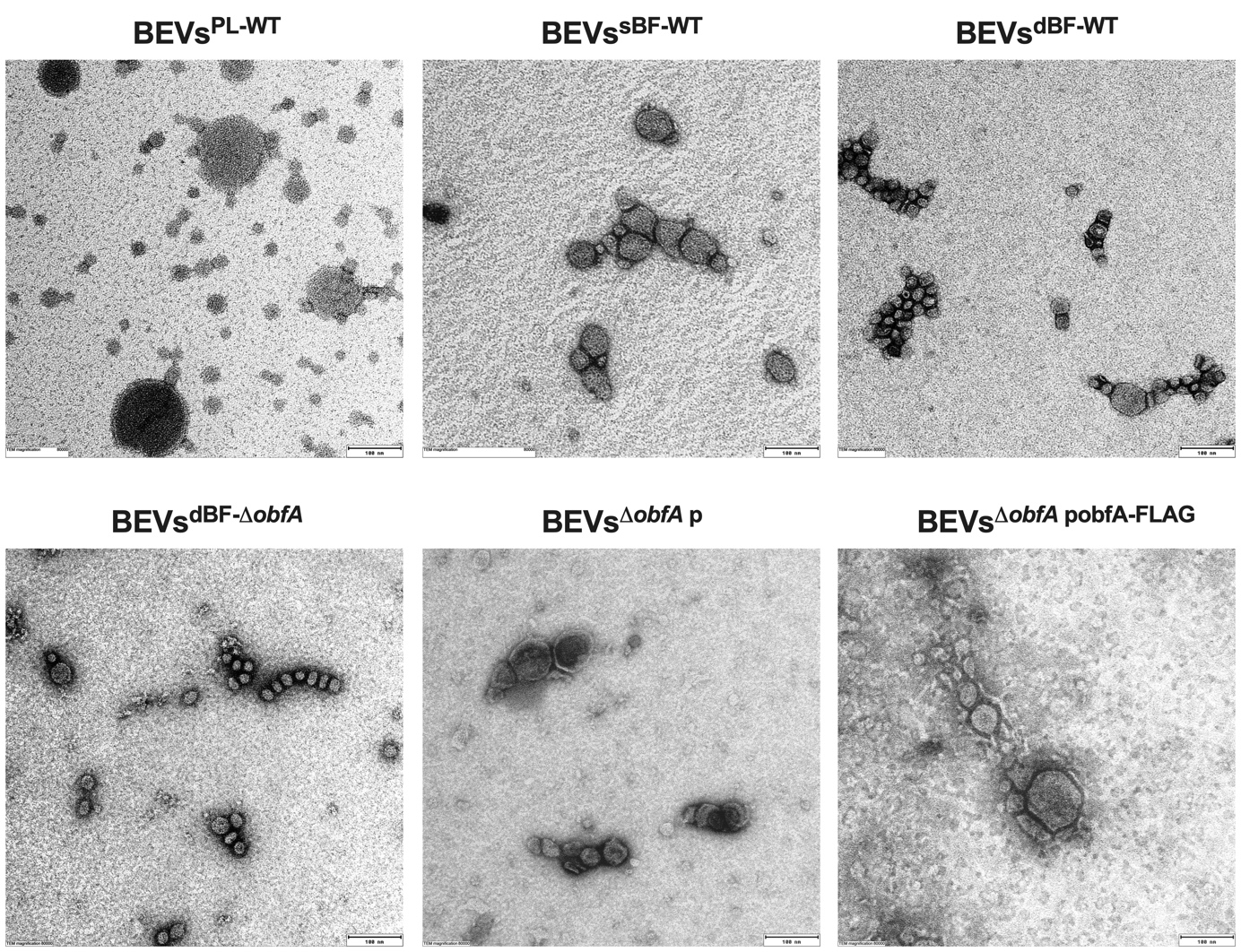
**

**Figure S2: Visualization of the isolated BEVs used in this study.** Shown are representative transmission electron microscopy (TEM) images visualizing the different BEVs used in this study. BEVs were isolated from *V. cholerae* WT planktonic cultures grown to late exponential phase (BEVs^PL-WT^), from *V. cholerae* WT mature static biofilms (BEVs^sBF-WT^), from *V. cholerae* WT or ∆*obfA* biofilms under dynamic flow conditions (BEVs^dBF-WT^ or BEVs^dBF-∆^*^obfA^*) as well as ∆*obfA* p and ∆*obfA* pobfA-FLAG planktonic cultures grown to late exponential phase with 0.5 mM IPTG. The scale bars represent 100 nm.


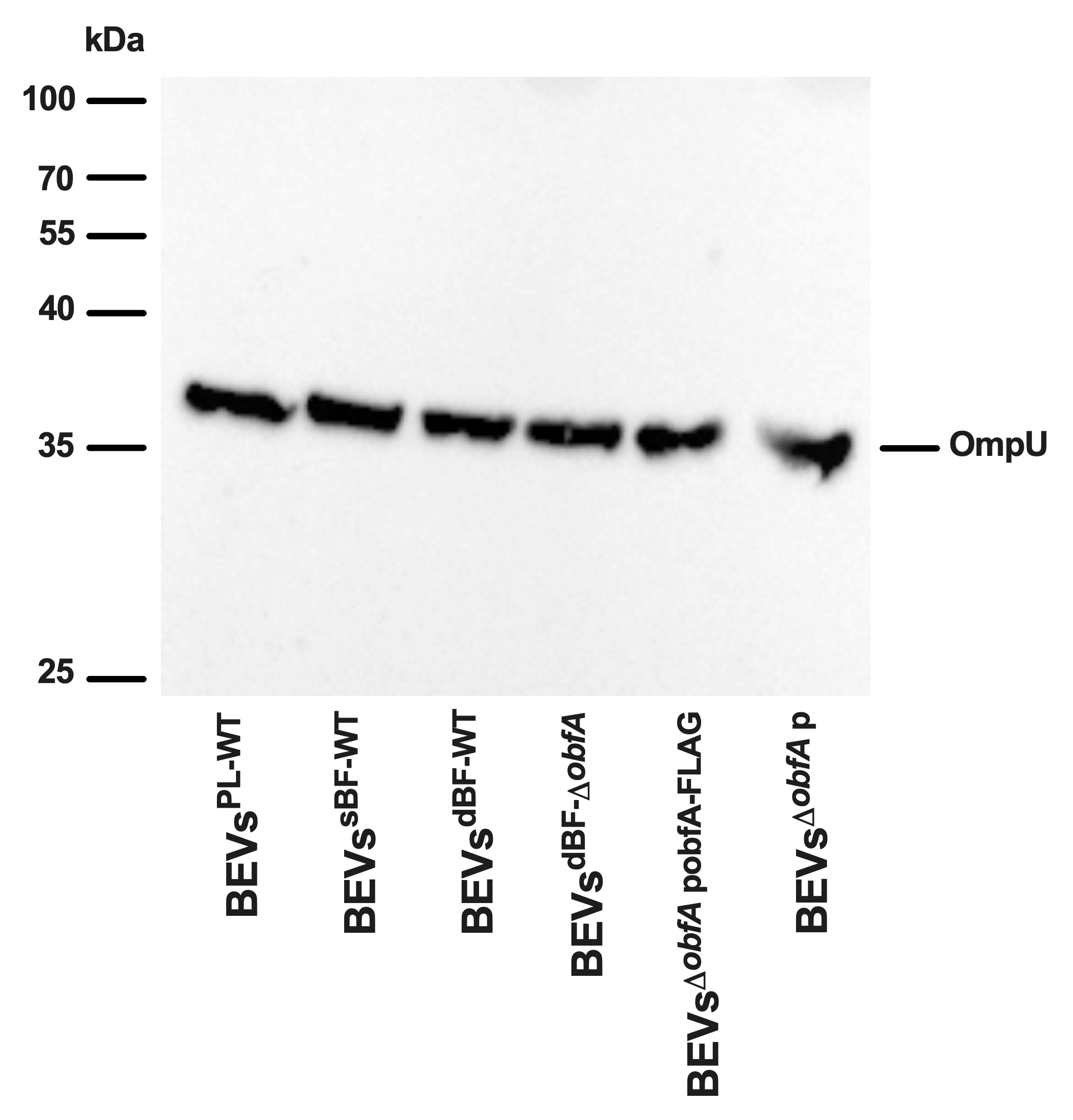


**Figure S3: Detection of OmpU in the isolated BEVs used in this study.** Shown is a representative immunoblot detecting the outer membrane protein OmpU (approx. 36 kDa) in BEVs isolated from *V. cholerae* WT planktonic cultures grown to late exponential phase (BEVs^PL-WT^), from *V. cholerae* WT mature static biofilms (BEVs^sBF-WT^), from *V. cholerae* WT or ∆*obfA* biofilms under dynamic flow conditions (BEVs^dBF-WT^ or BEVs^dBF-∆^*^obfA^*) as well as ∆*obfA* p and ∆*obfA* pobfA-FLAG planktonic cultures grown to late exponential phase with 0.5 mM IPTG. Approximately 5 µg protein equivalent of each sample were loaded onto the gels, separated by SDS-PAGE and further subjected to immunoblot analyses. Lines to the left indicate the molecular masses of the protein standards in kDa.

**
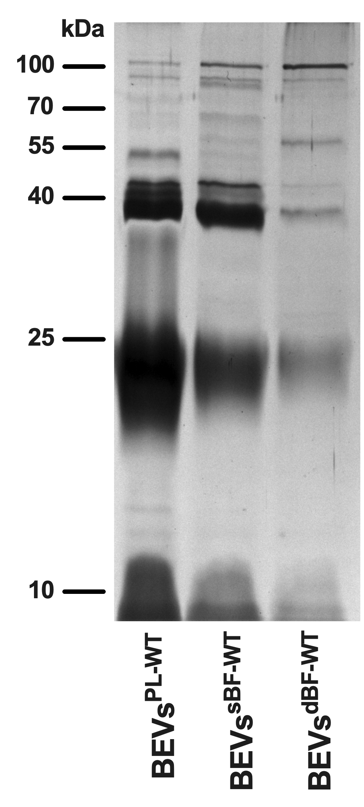
**

**Figure S4: Protein profiles of BEVs^PL-WT^, BEVs^sBF-WT^ and BEVs^dBF-WT^.** Shown are protein profiles of BEVs isolated from V. cholerae WT cultivated under planktonic (PL), static biofilm (sBF) and dynamic biofilm (dBF) conditions. Approximately 3 µg protein equivalent of each sample were loaded onto the gels, separated by SDS-PAGE and finally protein bands were visualized by silver staining. Lines to the left indicate the molecular masses of the protein standards in kDa.

**
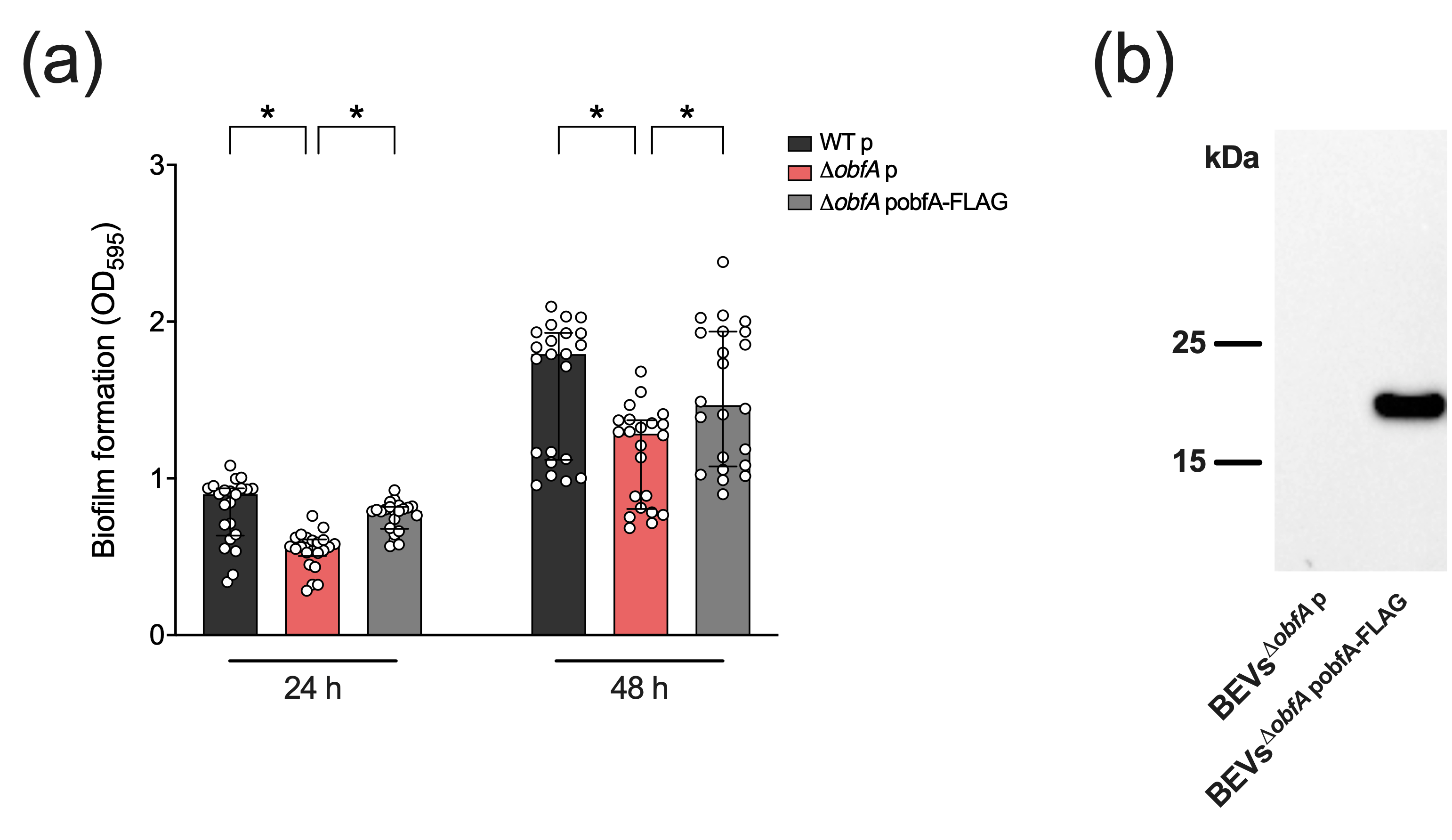
**

**Figure S5: Characterization of ObfA-FLAG expression strains.**

**(a)** Biofilms of WT and Δ*obfA* harbouring the empty vector (WT p and Δ*obfA* p) as well as Δ*obfA* pobfA-FLAG were quantified after 24 and 48 h in presence of 0.5 mM IPTG. The biofilm formation capacity was assayed under static conditions by crystal violet staining and subsequent determination of the OD_595_. Shown are the medians ± interquartile range (IQR) from 22 independent measurements (n = 22). An asterisk indicates a significant difference between the data sets (*, *P* < 0.05, using a Kruskal–Wallis test followed by *post hoc* Dunn's multiple comparisons).

**(b)** Shown is a representative immunoblot detecting the Flag-tagged ObfA in BEVs derived from Δ*obfA* pobfA-FLAG, while BEVs from Δ*obfA* harbouring the empty vector (Δ*obfA* p) served as negative control. BEVs were isolated from cultures grown to late exponential phase with 0.5 mM IPTG. Approximately 3 µg protein equivalent of each sample were loaded onto the gels. The commercially available anti-FLAG antisera specifically detecting the C-terminal FLAG-tag of the ObfA fusion construct was used for this immunoblot. Lines to the left indicate the molecular mass of the protein standard in kDa.

**
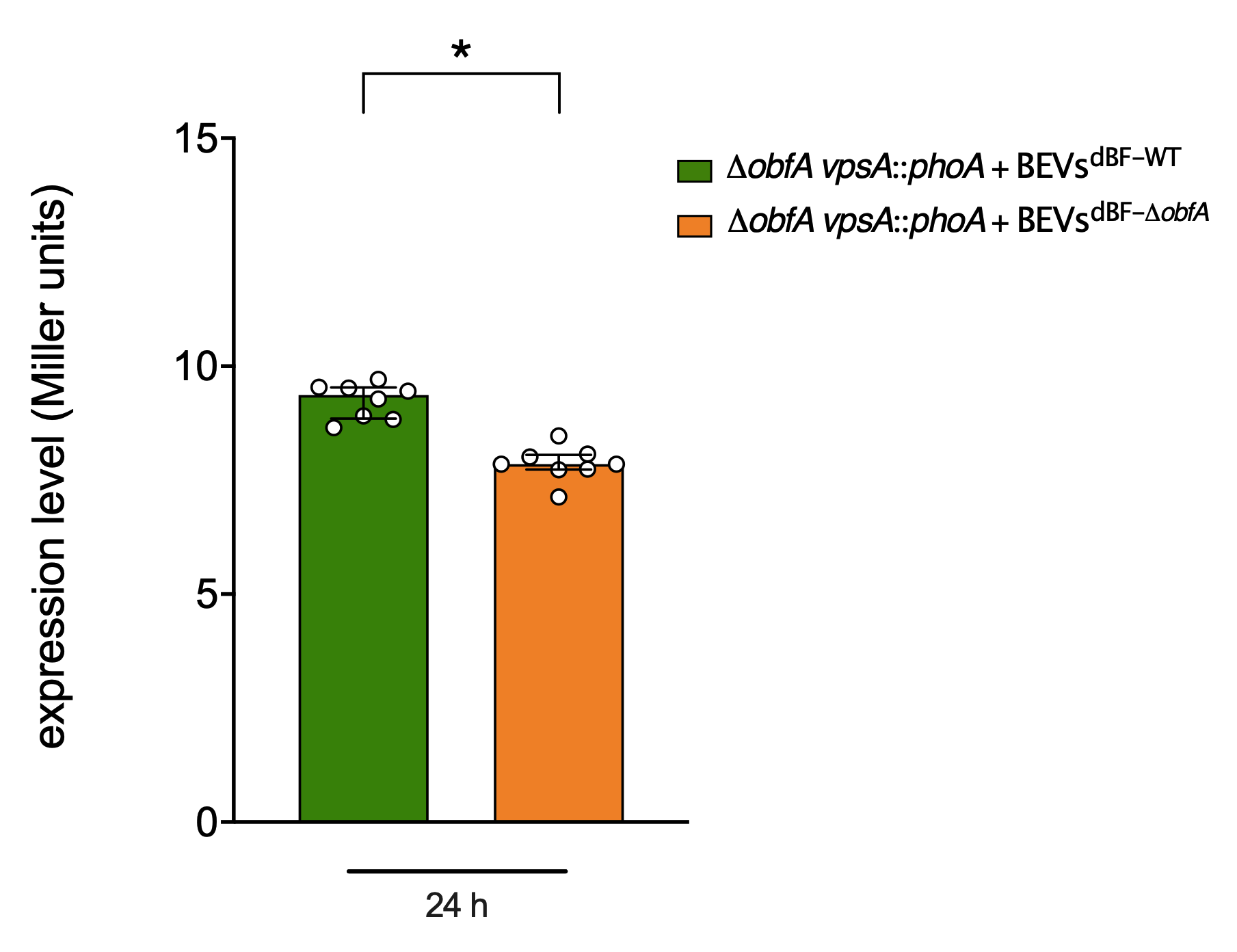
**

**Figure S6: Complementation of vpsA expression with isolated BEVs under dynamic conditions.** Alkaline phosphatase activities (in Miller units) were measured from a ∆*obfA* mutant harbouring a chromosomal *vpsA-phoA* transcriptional fusion. Cultures were grown at 24°C for 24 h. BEVs were isolated from WT (+BEVs^dBF-WT^) or ∆*obfA* (+BEVs^dBF-∆^*^obfA^*) cultures grown under dynamic biofilm conditions and added the biofilm assays at a final concentration of 0.02 μg µl^-1^. Shown are the medians ± IQR from eight independent measurements (n = 8). The asterisks indicate significantly different medians of the data sets (*, P < 0.05, using a Mann-Whitney U test).


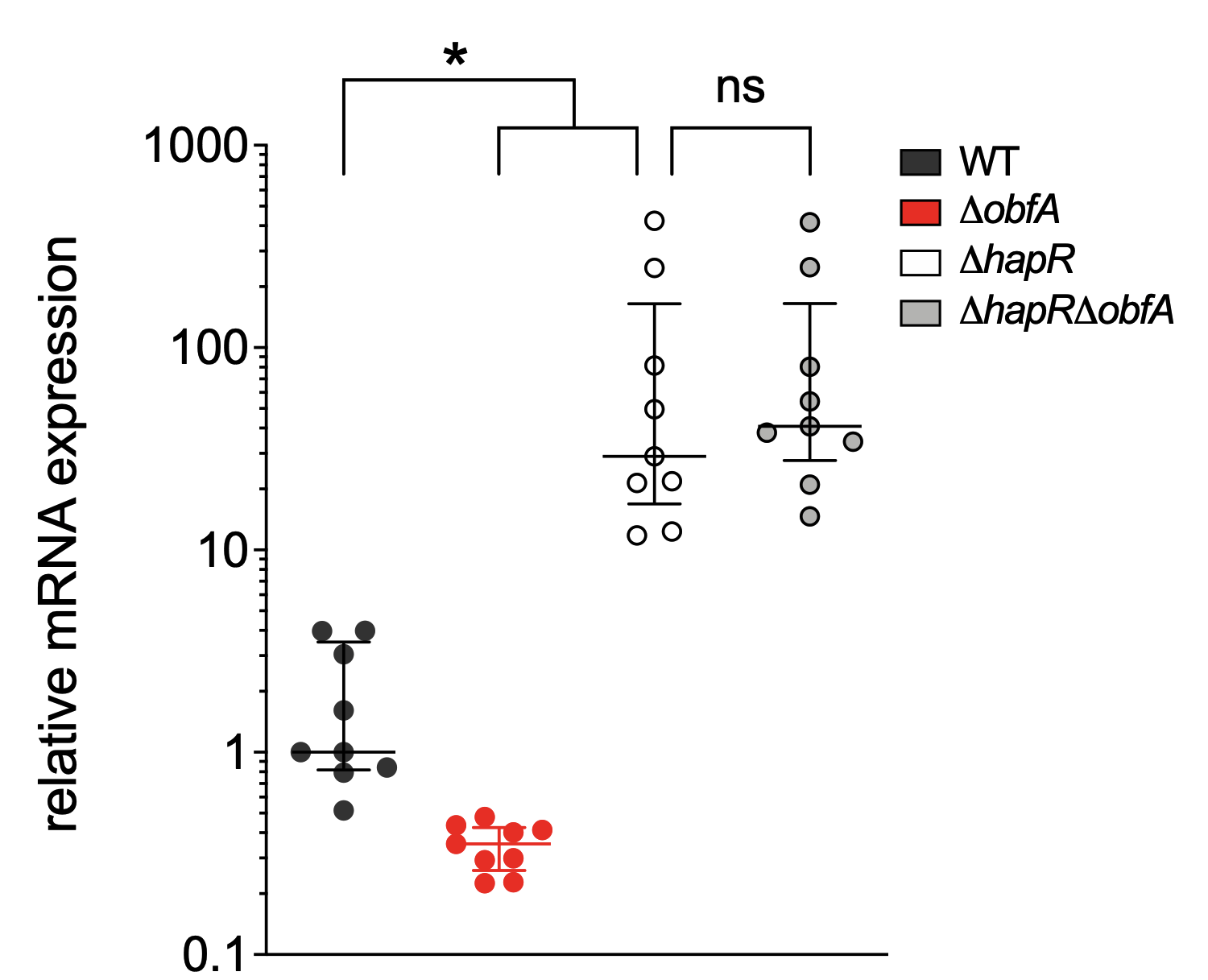


**Figure S7:** **Relative expression levels of *vpsA* in the WT and Δ*obfA*.** To assess expression levels bacterial RNA was extracted from cultures grown at 24°C for 48 h, reverse transcribed to cDNA and used as template for qRT-PCR analysis. For each sample, the mean cycle threshold of the test transcript was normalized to the housekeeping gene 16S rRNA and to one randomly selected WT reference sample. The data are presented as medians of nine independently grown samples (n = 9). An asterisk indicates a significant difference of the data sets, while ns indicates not significant differences (*, *P* < 0.05, using a Kruskal–Wallis test followed by *post hoc* Dunn's multiple comparisons).

**References**

1. Kolter R, Inuzuka M, Helinski DR. 1978. Trans-complementation-dependent replication of a low molecular weight origin fragment from plasmid R6K. Cell 15:1199-1208.

2. Roberts A, Pearson GD, Mekalanos JJ. Cholera vaccines strains derived from a 1991 Peruvian isolate of Vibrio cholerae and other El Tor strains, p. 43-47, p 43-47. *In* (ed),

3. Tamayo R, Schild S, Pratt JT, Camilli A. 2008. Role of cyclic Di-GMP during el tor biotype Vibrio cholerae infection: characterization of the in vivo-induced cyclic Di-GMP phosphodiesterase CdpA. Infect Immun 76:1617-27.

4. Seper A, Fengler VH, Roier S, Wolinski H, Kohlwein SD, Bishop AL, Camilli A, Reidl J, Schild S. 2011. Extracellular nucleases and extracellular DNA play important roles in Vibrio cholerae biofilm formation. Mol Microbiol 82:1015-37.

5. Donnenberg MS, Kaper JB. 1991. Construction of an *eae* deletion mutant of enteropathogenic *Escherichia coli* by using a positive-selection suicide vector. Infect Immun 59:4310-4317.

6. Roier S, Zingl FG, Cakar F, Durakovic S, Kohl P, Eichmann TO, Klug L, Gadermaier B, Weinzerl K, Prassl R, Lass A, Daum G, Reidl J, Feldman MF, Schild S. 2016. A novel mechanism for the biogenesis of outer membrane vesicles in Gram-negative bacteria. Nat Commun 7:10515.

7. Svenningsen SL, Waters CM, Bassler BL. 2008. A negative feedback loop involving small RNAs accelerates Vibrio cholerae's transition out of quorum-sensing mode. Genes Dev 22:226-38.

8. Henke JM, Bassler BL. 2004. Three parallel quorum-sensing systems regulate gene expression in Vibrio harveyi. J Bacteriol 186:6902-14.

9. Amann E, Ochs B, Abel KJ. 1988. Tightly regulated *tac* promoter vectors useful for the expression of unfused and fused proteins in *Escherichia coli*. Gene 69:301-315.

10. Moisi M, Jenul C, Butler SM, New A, Tutz S, Reidl J, Klose KE, Camilli A, Schild S. 2009. A novel regulatory protein involved in motility of Vibrio cholerae. J Bacteriol 191:7027-38.
